# Probing Degradation in Lithium Ion Batteries with On‐Chip Electrochemistry Mass Spectrometry**

**DOI:** 10.1002/anie.202315357

**Published:** 2023-12-29

**Authors:** Daisy B. Thornton, Bethan J. V. Davies, Soren B. Scott, Ainara Aguadero, Mary P. Ryan, Ifan E. L. Stephens

**Affiliations:** ^1^ Department of Materials Imperial College London London SW7 UK; ^2^ The Faraday Institution Harwell Science and Innovation Campus Harwell OX11 0RA UK

**Keywords:** Degradation, Electrochemistry, Gas Evolution, Lithium Ion Batteries, Mass Spectrometry

## Abstract

The rapid uptake of lithium ion batteries (LIBs) for large scale electric vehicle and energy storage applications requires a deeper understanding of the degradation mechanisms. Capacity fade is due to the complex interplay between phase transitions, electrolyte decomposition and transition metal dissolution; many of these poorly understood parasitic reactions evolve gases as a side product. Here we present an on‐chip electrochemistry mass spectrometry method that enables ultra‐sensitive, fully quantified and time resolved detection of volatile species evolving from an operating LIB. The technique's electrochemical performance and mass transport is described by a finite element model and then experimentally used to demonstrate the variety of new insights into LIB performance. We show the versatility of the technique, including (a) observation of oxygen evolving from a LiNiMnCoO_2_ cathode and (b) the solid electrolyte interphase formation reaction on graphite in a variety of electrolytes, enabling the deconvolution of lithium inventory loss (c) the first direct evidence, by virtue of the improved time resolution of our technique, that carbon dioxide reduction to ethylene takes place in a lithium ion battery. The emerging insight will guide and validate battery lifetime models, as well as inform the design of longer lasting batteries.

## Graphical abstract

In batteries, the formation of gases such as oxygen, carbon dioxide and ethylene accompanies degradation. Ultrasensitive on chip electrochemistry mass spectrometry reveals previously undetectable gas evolution in lithium ion batteries. The ensuing insight will enable battery scientists to predict degradation mechanisms and discover new strategies to stabilise device performance.

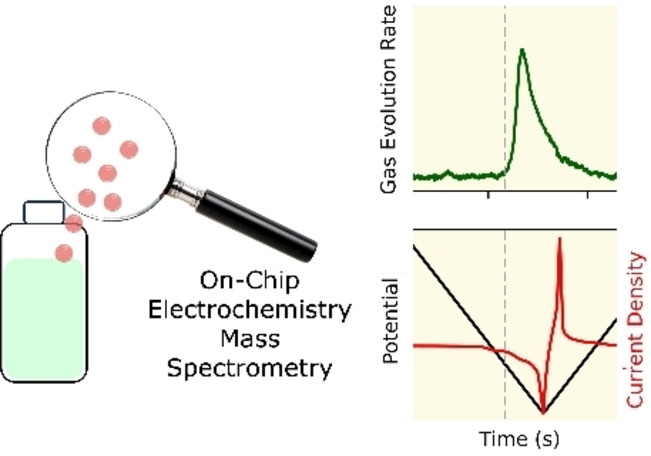

## Introduction

The continued concerns and challenges of climate change, environmental pollution and the depletion of fossil fuels have motivated research to develop high performance lithium ion batteries that enable the electrification of transport.[^1^, ^2^, ^3^, ^4^] In order to accelerate this vital electrification, optimisation of cell chemistry is vital. In particular improvements to capacity and longevity are required to address “range anxiety” and reduce overall battery cost, allowing EVs to reach cost parity with conventional gasoline vehicles.

The cathode material LiNi_x_Mn_y_Co_z_O_2_ (NMC) is of particular interest as it is able to take advantage of the synergistic effects provided by a transition metal mixture.[^5^, ^6^] The ratio of nickel to manganese to cobalt has significant implications on the electrochemical performance of an NMC containing electrochemical cell. When the nickel content of the transition metal oxide is increased, the capacity increases. Similarly, the cell cost is reduced as the fraction of unethically sourced, expensive cobalt required is decreased. Unfortunately, this capacity improvement requires high voltages and a high extent of delithiation, which results in degradation via a variety of mechanisms such as structural phase transitions,[^7^, ^8^, ^9^] electrolyte decomposition,[^10^, ^11^, ^12^] and transition metal dissolution,[^13^, ^14^, ^15^] for example. These degradation mechanisms cause decreased electrochemical performance and safety as a function of cycling, leading to batteries with much shorter lifetimes. Unfortunately though, the precise mechanisms by which these reactions take place remain poorly understood, which limits our ability to mitigate their effects.

**Figure 1:**
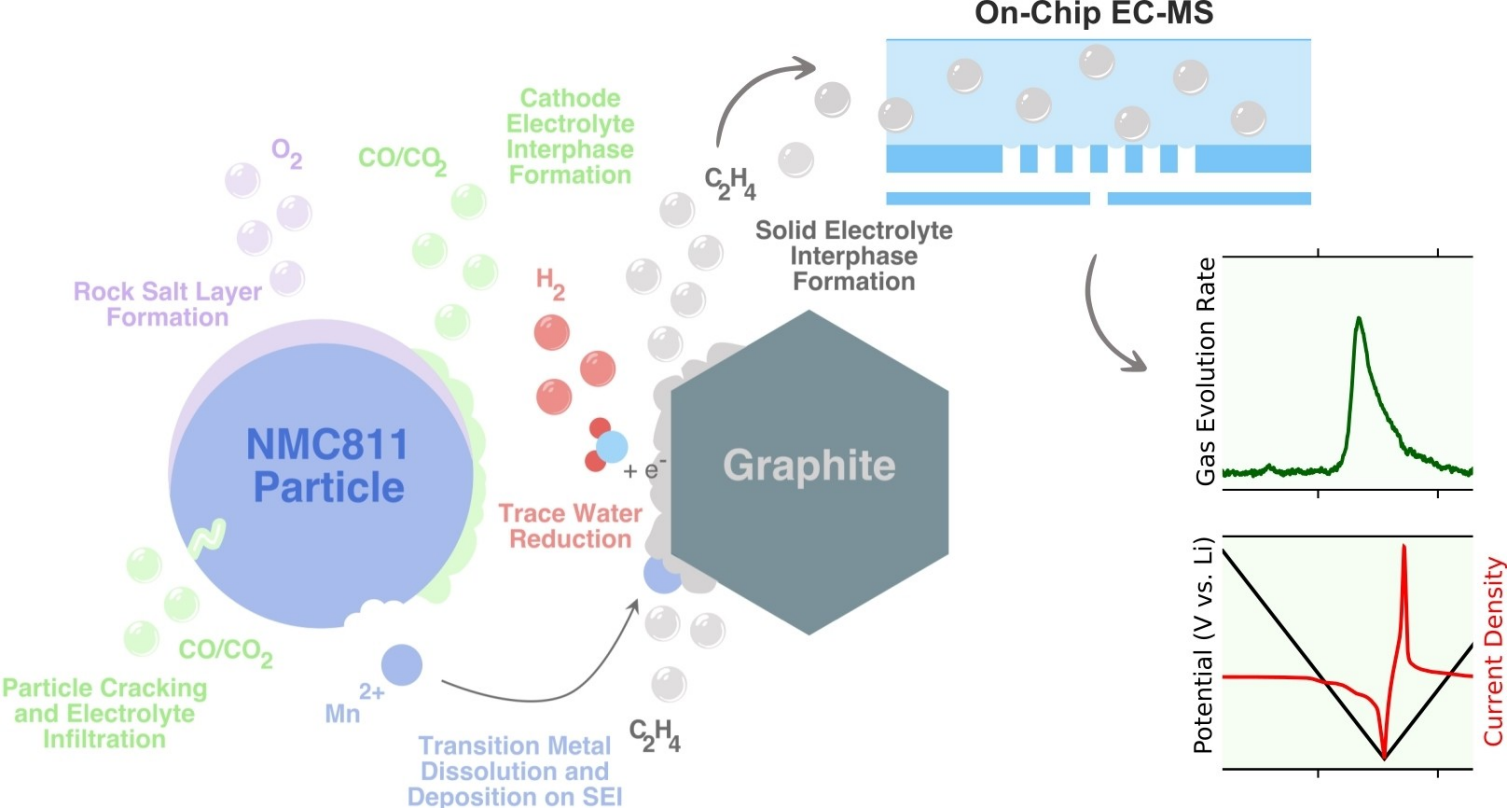
Schematic showing a variety of gas evolving parasitic reactions and degradation mechanisms that can take place in a LiNi_0.8_Mn_0.8_Co_0.1_O_2_ (NMC811) based lithium ion battery. On‐chip EC‐MS can sensitively monitor the evolution of these volatile species as a function of electrochemical cycling with improved time resolution, providing valuable insight, facilitating the development of better batteries.

Many of the degradation mechanisms that contribute to the declining health of NMC containing LIBs are accompanied by some form of gas evolution shown in Figure 1. For example, the highly reactive charged surface of an NMC particle catalyses the decomposition of the electrolyte, depositing a solid interphase layer on the electrode surface known as the cathode electrolyte interphase (CEI).^[16]^ Similarly, such a layer is formed on the graphite electrode where it is known as the solid electrolyte interphase (SEI).^[17]^ Although these layers contribute some beneficial properties (in fact the SEI on the anode is required for stable electrochemical performance in a wide potential window), if they become too thick, they can impede lithium mobility or trap active lithium, depleting the reservoir of ions able to contribute to the cell redox and reducing the discharge capacity. Example SEI and CEI formation reactions can be seen in Equation 1 (note O* denotes a surface species and * denotes a vacant surface site). The SEI formation reaction shows the reduction of ethylene carbonate to ethylene and lithium diethyl carbonate (LEDC). The CEI formation reaction shows the reaction of ethylene carbonate with surface oxygen forming CO, CO_2_, H_2_O and oxygen vacancies. The equations show that along with the formation of the resistive and lithium containing solid interphase components, these reactions form volatile species such as C_2_H_4_, H_2_, O_2_, CO_2_ and CO. The ability to sensitively and precisely pinpoint the formation and evolution of the volatile species as a function of electrochemical cell operation will provide valuable insight into these complex degradation mechanisms. The ensuing understanding can contribute to the development of mitigation strategies and the ability to better model and predict battery lifetimes.
(1)

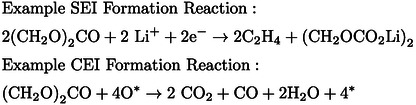




Although we know that the SEI should provide good Li^+^ conductivity whilst also being electronically insulating, the composition and morphology that is best able to provide these properties is still under debate.[^16^, ^18^, ^19^, ^20^, ^21^] For example, Zhang et al. hypothesise that the SEI component lithium ethyl dicarbonate (LEDC) forms a stable SEI due to its ability to form an organic network,^[22]^ whereas Lucht and co‐workers have proposed that LEDC decomposes to a passivating Li_2_CO_3_ film in the SEI.^[21]^

Similarly at the NMC electrode we know that oxygen release, electrolyte oxidation and the deposition of decomposition products takes place.[^12^, ^23^, ^24^, ^25^] These processes are highly interlinked but poorly understood. Notably, oxygen evolution is not usually observed in most gassing measurements[^8^, ^26^] so it is often assumed that a highly reactive oxygen species is formed^[12]^ and chemically reacts with the electrolyte solvent to form CO_2_ and CO. Østergaard et al. proposed a surface reaction of the organic electrolyte that leads to the removal of oxygen and the formation of CO_2_ and CO, in contrast to the mechanism that forms an oxygen molecule.^[24]^

Identifying and quantifying gas evolution during the operation of an electrochemical system provides valuable insight into a variety of important processes. Example areas where such insight is useful in the LIB field have been briefly mentioned above, however these measurements are of significant use for many electrochemistry disciplines—from determining the activity of a catalyst to quantifying competing reactions. Performing such measurements accurately usually requires electrochemistry mass spectrometry (EC‐MS) to interfaces the liquid environment of an electrochemical cell with the vacuum environment of a mass spectrometer.^[27]^ The two most commonly used techniques are Differential Electrochemical Mass Spectrometry (DEMS) and Online Electrochemical Mass Spectrometry (OEMS).[^28^, ^29^, ^30^, ^31^, ^32^] More recently, a new technique has been developed, known as On‐Chip Electrochemistry Mass Spectrometry (EC‐MS), which overcomes a variety of limitations of DEMS and OEMS.^[33]^ Notably, EC‐MS improves the sensitivity and time‐resolution to detect volatile species, providing a very valuable tool for investigating parasitic reactions.

The technique was originally developed for aqueous electrochemistry and has been described and compared to other techniques in more detail by Trimarco et al.^[33]^ On‐chip EC‐MS has improved sensitivity and time resolution compared to other EC‐MS techniques such as OEMS and DEMS. For example, on‐chip's sensitivity is of the order of picomoles per second, whereas the DEMS and OEMS sensitivity is of the order of nanomoles. Similarly, on‐chip EC‐MS has sub‐second time resolution, whereas the time resolution in DEMS and OEMS is on the order of seconds. Therefore, by applying on‐chip EC‐MS to study lithium ion batteries, new insight can be gained on parasitic gassing reactions. Herein, we adapt the technique for non‐aqueous systems: in particular we redesign the cell to account for the lower conductivity of organic electrolytes, as well as develop new experimental and analytical protocols. We achieve highly sensitive and time resolved measurements of gas evolution from non‐aqueous electrochemical systems (such as lithium ion batteries) without compromising the electrochemical performance of the system being investigated. To the best of our knowledge, the only other published non‐aqueous application of on‐chip EC‐MS is electrochemical nitrogen reduction to ammonia.^[34]^ Although the work in this area looks extremely promising, the use of a cell design tailored for non‐aqueous applications may facilitate further development.

We demonstrate the value of the technique development by gaining new insight into both graphite anode and nickel‐rich NMC cathode degradation mechanisms. In particular, the relationship between oxygen release from NMC and the oxidative decomposition of the electrolyte solvents is studied. We focus on exploring which degradation pathways contribute the most to the observed gassing, developing an understanding of how impurities and various electrolyte solvents affect cell degradation. At the graphite anodes, we similarly explore how various electrolyte solvents contribute to initial SEI formation—does ethylene carbonate reduction dominate? Further, we demonstrate the technique's ability to simulate cross‐talk reactions, gaining insight into how CO_2_ that is evolved at the cathode might react and influence the anode's SEI properties.

## Results and Discussion

### Electrochemical Cell Design

Coupling the EC‐MS analytical system with a well‐designed electrochemical cell is of vital importance for accurate interpretation of the mass spectrometry measurements. On‐chip EC‐MS is generally coupled with a stagnant thin layer electrochemistry cell that fixes the working electrode parallel to and 100 μm from the membrane surface. Having the working electrode facing the membrane facilitates fast collection of all volatile species from the cell. Placing the working electrode at a known distance from the membrane also fully defines the mass transport of the volatile species as they diffuse towards the membrane where they evaporate into the chip sampling volume. As mentioned above, the EC‐MS system allows reactant gases to be introduced to the electrochemical cell through the chip, where they dissolve into the electrolyte from the membrane and diffuse towards the working electrode. The fixed and known working electrode position therefore facilitates both fast transport of reactant and product gases to and from the working electrode.

The high conductivity of aqueous electrolytes means the performance of the working electrode is relatively insensitive to the exact position of the counter electrode. The relatively poor ionic conductivity of non‐aqueous electrolytes (such as LIB electrolytes for example) means that cell electrode positioning becomes important, with conductivities at least an order of magnitude smaller than aqueous systems.^[35]^ This effect can be so extreme that even a slight misalignment of parallel electrodes in a LIB coin cell results in a non‐uniform current distribution and non‐reproducible electrochemical behaviour.^[36]^ Therefore it is clear that the EC‐MS aqueous cell, with its side by side electrodes, is unsuitable for LIB studies as it will lead to highly heterogeneous current distributions. This effect was confirmed by building a 2‐dimensional finite element method model with COMSOL Multiphysics (5.6) (see Section 4 in the SI), reiterating the need to redesign the cell for use with non‐aqueous electrolytes.

**Figure 2:**
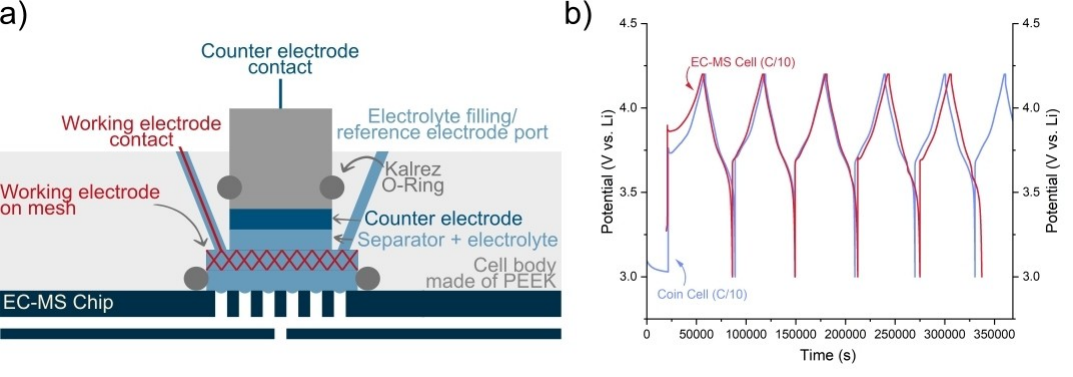
a) A conceptual diagram showing the new non‐aqueous electrochemical cell mounted on top of the EC‐MS chip. The central hole contains the counter electrode, a separator wetted by the electrolyte, followed by the mesh current collector coated in the working electrode slurry and contacted through a port. Another more porous separator is located between the working electrode and the EC‐MS chip and wetted by the electrolyte, facilitating the fast, downward diffusion of evolved gases towards the chip and mass spectrometer, without compromising electrochemical performance. b) An electrochemical comparison between the new non‐aqueous EC‐MS cell and a research standard coin cell, cycling NMC811 (WE) *vs*. Li (CE) at C/10, showing comparable performance.

Here, we present an optimised cell design that enables sensitive and time resolved operando gas evolution measurements in LIBs (and other non‐aqueous electrochemical systems) without compromising cell performance, inspired by cells used for operando spectroscopy techniques. Figure 2 a) conceptually demonstrates the operating principle of the new cell design. The cell body is made of PEEK and has a central hole in which the electrode stack is placed. The design employs a working electrode at the bottom of the central hole that is contacted to a circular expanded metal (flat mesh) current collector. This enables the working electrode to face the counter electrode while simultaneously allowing gasses to pass through the current collector and diffuse towards the membrane chip. There is a 100 μm thick, porous separator (Celgard 2500), wetted by the electrolyte, in between the membrane chip and working electrode to keep the working electrode in place at a fixed distance and to ensure the electrode is fully wetted by the electrolyte. Above the working electrode is a denser or thicker separator (Celgard 2325 or Whatman glass microfibre GF/C), which prevents short circuit with the counter electrode and forces gases to diffuse downwards towards the membrane chip through the more porous separator and prevent cross‐talk. The working electrode may be deposited directly on to the current collector or on to either separator. Above the denser separator is the counter electrode, electrically contacted by a plunger. The plunger also has an O‐ring fitted to ensure the cell can be fully sealed from atmosphere. The working electrode current collector is electrically contacted through a sealed port. The cell contains another port which may be used for electrolyte filling or may contain a reference electrode if required. At the bottom of the cell, an O‐ring seals against the membrane chip, which is connected to the mass spectrometer and held in place by a clamping ring. More experimental details of the cell assembly and data collection may be found in the Section 1 of the Supporting Information. More detailed computer aided design Figures can be seen in Section 3 of the SI.

Our finite element modelling (see Section 4 in the SI) shows that the new cell design facilitates heterogeneous state of charge and potential distribution, whereas the original aqueous cell does not. Figure 2b) shows a comparison of the new non‐aqueous EC‐MS cell's electrochemical performance to a research standard coin cell built with the same sized working electrodes. The cells were built with a NMC811 coated mesh electrode versus lithium metal (providing an excess of lithium in the cell). The EC‐MS cell performs very well when compared to the coin cell: the potential profiles are almost identical confirming the excellent suitability of the new EC‐MS cell for studying realistic and relevant electrochemical parasitic reactions in lithium ion batteries. Electrodes from commercial cells, including aged cells could also be tested in a strip configuration, as described in Section 3 in the SI.

### On‐Chip Electrochemistry Mass Spectrometry for Lithium Ion Batteries Results

#### NMC vs. Li

**Figure 3:**
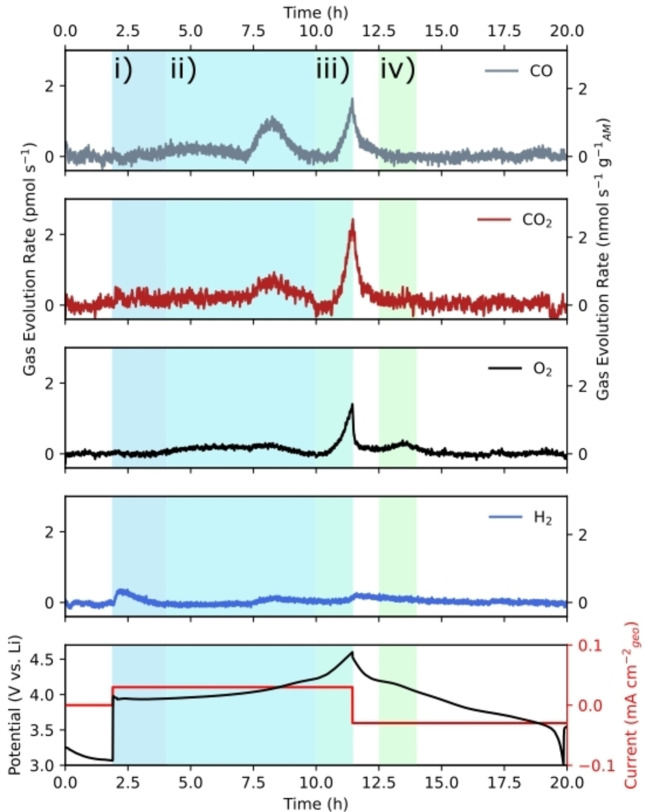
Quantified gas evolution rates (in pmol per second and nmol per second per gram of active material) of CO, CO_2_, O_2_ and H_2_ during the first cycle of NMC811 (WE) *vs*. Li (CE) in 1 M LiPF_6_ in EC/EMC (3 : 7), cycled at C/10 as a function of time. The measurements were carried out at room temperature.

The fully quantified H_2_, CO, CO_2_ and O_2_ evolution from the first formation cycle in a NMC *vs*. Li cell is shown in Figure 3. The data processing and quantification details are given in the SI. The cell was cycled at a rate of C/10 from open circuit voltage to 4.6 V_Li_ and back to 3.0 V_Li_. Four gas evolution processes are distinguished: i) on application of current, ii) during charge, iii) at high potential, and iv) on discharge.

Firstly upon application of current and initial polarisation of the cell, hydrogen is observed at ≈3.9 V_Li_ and peaks at a rate of 0.4 nmol s^−1^ g^−1^_active material_. We hypothesise that this hydrogen originates from trace water reduction reactions in the electrolyte (Equation 2).

At higher potentials above 4 V_Li_, H_2_ evolves again, coinciding with CO_2_ and CO evolution. The CO_2_ and CO are related to the oxidation of the organic electrolyte solvents (EMC and EC) at the cathode. Non volatile, protic organic species may also generated through these processes, cross over to the anode and reduce to form H_2_ (Equation 3). H_2_ evolution is also observed in region ii and iii and likely coincides with the generation of protic species at the cathode and resulting cross‐talk and reduction at the anode.^[37]^ This is further evidenced by the observation of electrolyte or surface species generating CO and CO_2_ at the cathode in the same regions, which we discuss below.

(2) $$H_{2}O+e^{-}\to 0.5H_{2}+OH^{-}$$

(3) $$2RH+2e^{-}\to H_{2}+2R$$

A weak oxygen evolution signal is observed from 3–10 hours. This may be related to the electrochemical oxidation of lithium carbonate contamination of the cathode. The lithium carbonate is present as a result of the cathode material synthesis and exposure to air and moisture during preparation of the cathode slurry which is performed outside of the glovebox.^[14]^

During the initial charge of NMC cathode, lithium carbonate on the surface is removed.^[38]^ The mechanism of this removal is still debated. The two current hypotheses are electrochemical oxidation and chemical degradation.^[38]^ Although the chemical degradation pathway is observed to be potential dependent, occurring around 3.8 V vs. Li the degradation is caused by acidification of the electrolyte due to oxidation of organic electrolyte components at the cathode at these potentials and above (Equation 4). The electrochemical oxidation mechanism (Equation 5) would release O_2_ as well as CO_2_. The observation of oxygen evolution in region ii may be a result of the electrochemical oxidation of lithium carbonate on the surface. However, detailed analysis of this process is beyond the scope of this article and is the focus of a study underway.

In region ii and iii, CO and CO_2_ evolve concomitantly. In region ii broad peaks are observed when charging from 4.0 to 4.2 V_Li_ and a sharper, more symmetrical peak is observed around the high voltage region at 4.6 V_Li_. Prior reports of the lower potential region (i) have attributed CO_2_ evolution to the removal of adventitious Li_2_CO_3_, typically occurring at or above 3.8 V_Li_, either by chemical dissociation (Equation 4) or electrochemical oxidation (Equation 5) routes.^[38]^ The concurrent observation of O_2_ in region iii indicates that the latter reaction may play a role. However, the additional observation of CO, which is not a product of either of these routes, suggests that this peak is, at least in part, related to the oxidation of the organic solvent carbonates EC and EMC. A subsequent process occurs at high voltage, which also generates both CO and CO_2_ at a maximum rate of 1.7 and 2.5 nmol s^−1^ g^−1^_active material_ respectively.

(4) $$Li_{2}CO_{3}+{2H}^{+}\to 2\;Li^{+}+CO_{2}+H_{2}O$$

(5) $$2{\mathrm{Li}}_{2}{\mathrm{CO}}_{3}\to 4{\mathrm{Li}}^{+}+2{\mathrm{CO}}_{2}+O_{2}+4e^{-}$$

The total amounts of the generated gases were calculated and we obtained a ratio of approximately 1.4 : 2.0 for CO : CO_2_, which is comparable to often reported values.[^39^, ^40^] A 1 : 2 ratio would correspond to the oxidation pathway of ethylene carbonate (as shown in Equation 6 or Equation 7). EMC is also present in the electrolyte (70 % wt, compared to EC). EMC oxidation takes place via Equation 8 and should generate a 1 : 1 ratio of CO to CO_2_, therefore the observed ratio of 1.4 : 2 indicates oxidation of both solvent components. The precise quantification of the total amounts of gases evolved provides insight into the electrolyte oxidation reactions that take place at the NMC cathode, highlighting its reactivity to both EC and EMC.

(6) $${\left(CH_{2}O\right)}_{2}CO+2O_{2}\to 2\;CO_{2}+CO+2H_{2}O$$

(7)

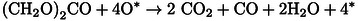

(8) $$CH_{3}CH_{2}OCOOCH_{3}+O_{2}\to CH_{3}CH_{2}OH+CO_{2}+CO+H_{2}O$$

Lastly, we will discuss the observation of oxygen evolution in our system. The mechanism and effect of oxygen formation in the NMC cell is a disputed process.[^8^, ^12^, ^41^, ^42^, ^43^] Due to the lack of observation of oxygen in many gassing measurements[^8^, ^26^] it is often assumed that a highly reactive oxygen species is formed^[12]^ reacting with organic electrolyte components to generate CO_2_ and CO. The EC‐MS setup is able to sensitively detect O_2_ evolution from the cell, meaning that no assumptions need to be made, so that we can decisively pinpoint the origin of the CO_2_ and CO. Although some experimental techniques are able to observe O_2_ evolution from NMC,[^12^, ^44^] the amounts evolved are very close to the detection limit. The demonstrated sensitivity of the EC‐MS means that even as we move towards the future, more stable cathode materials, it will remain relevant for detecting degradation via oxygen release. Based on our measurements within this manuscript, the origin of the oxygen cannot be confirmed. However, based on literature, NMC materials release oxygen at high states of charge when transitioning from a layered oxide to a rock salt structure.[^12^, ^40^, ^46^]

Østergaard et al.^[24]^ suggested a surface reaction with the electrolyte solvent causing the removal of oxygen, notably a different route to the more commonly proposed mechanism that involves the formation of molecular oxygen. Distinguishing between the highly reactive molecular oxygen release and the surface reaction is of importance as it will guide the development of mitigation strategies to prevent this harmful degradation mechanism. We present here the observation of both molecular oxygen and CO_2_. This demonstrates the detection ability of the EC‐MS and we are conducting further studies to better answer where the oxygen originates from and how it reacts with the organic carbonate.^[45]^

In region ii we observe a small and broad peak in O_2_ evolution, related to the H2‐H3 phase change.^[46]^ In region iii, at high state of charge, we observe a sharp asymmetrical peak in O_2_ evolution. The high time‐resolution of the EC‐MS system allows us to observe different rates of formation of CO_2_/CO and O_2_. A precipitous drop in the O_2_ signal on discharge but not in the CO_2_ and CO signals in region iii indicates a potential dependent oxidation of solvent to form CO_2_ and CO, with O_2_ evolution more related to the state of charge and structural changes. The effect of the phase change is more clearly observed in the gas flux vs. potential plot (Figure 12 in SI). Notably, another O_2_ peak is again observed on the discharge in region iv and is related to a structural change in NMC mirroring the reverse H3‐H2 phase change as observed on charge. This gives valuable insight into the oxygen release mechanism as it highlights that oxygen is more mobile in the NMC structure at specific states of lithiation, rather than just after a certain state of charge has been reached.

#### Graphite vs. Li

**Figure 4:**
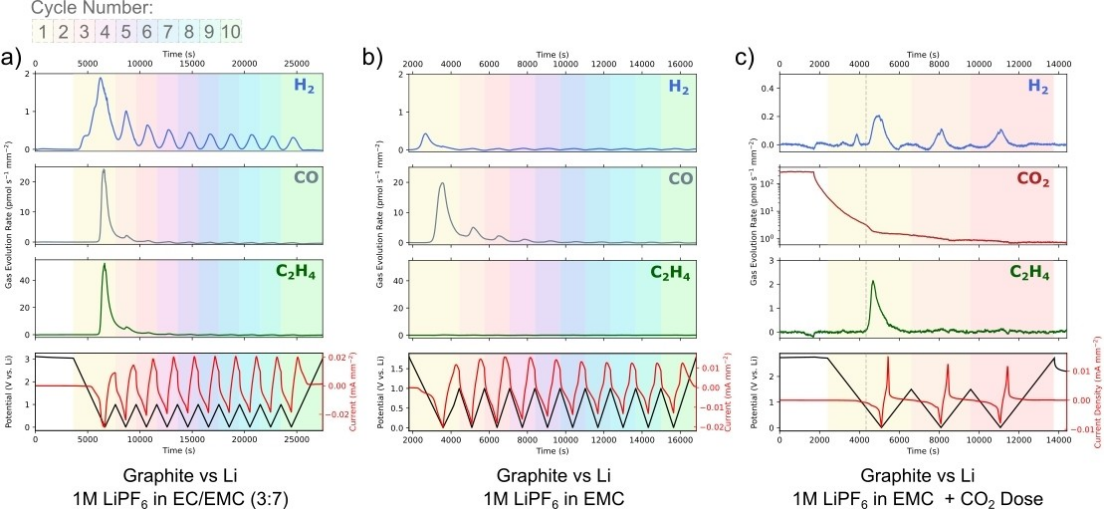
Electrochemistry mass spectrometry data showing the measured H_2_, CO and C_2_H_4_ signals as well as the potential and current during cyclic voltammetry (0.01–1 V *vs*. Li, 1 mV s^−1^) in graphite (WE) *vs*. Li (CE) cells using a) 1 M LiPF_6_ in EC/EMC (3 : 7) electrolyte and b) 1 M LiPF_6_ in EMC electrolyte. c) The measured H_2_, CO_2_ and C_2_H_4_ signals during cyclic voltammetry (0.01–1 V *vs*. Li, 1 mV s^−1^) in a graphite *vs*. Li that has been dosed with CO_2_ at OCV, showing direct evidence of CO_2_ reduction to C_2_H_4_ in an operating LIB. Note the different axis scales on c).

Figure 4a) shows the measured and fully quantified H_2_, CO and C_2_H_4_ gas evolution from a graphite *vs*. Li cell in LP57, cycled between 0.01 and 1 V_Li_ for 10 cycles. H_2_ evolution is observed at a maximum rate of 1.9 pmol s^−1^ mm^−2^ in the first cycle. The general consensus in the literature is that any hydrogen evolved at the anode is due to the reduction of trace moisture present in the cell.[^37^, ^39^, ^48^] However, the highly time resolved method presented in this work is able to differentiate numerous processes contributing to H_2_ evolution, as seen by the multiple peaks resolved in the first cycle. These processes are potential dependent, with peaks observed to begin at 2.5, 1.5 and 1 V_Li_. Based off previous works by Novak et al. we assign these peaks to the electrochemical reduction of H_2_O (2.5 and 1.5 V_Li_) and SEI formation (1 V_Li_).^[29]^ The H_2_ evolution accompanying SEI formation is likely a product of H‐containing surface groups combining or electrolyte solvent decomposition. At ≈0.9 V_Li_ H_2_ evolution stops, coinciding with the onset of C_2_H_4_ and CO evolution. This effect is observed in the second cycle as well and suggests that the SEI forming reaction that evolves H_2_ competes with the reactions that form C_2_H_4_ and CO. The competition of the reactions forming H_2_ and those forming C_2_H_4_ and CO could be due to competing adsorption e.g. CO* versus H* at surface sites, potentially due to changes in the local activity of the reactant molecules adjacent to the electrode.

In the first cycle at potentials below 0.9 V_Li_, mostly C_2_H_4_ (around 55 pmol s^−1^ mm^−2^ in the first cycle) is observed. Earlier reports have shown that ethylene evolves from the reductive decomposition of the ethylene carbonate (EC) solvent.[^18^, ^19^, ^22^] This decomposition forms one of two SEI components: lithium ethyl dicarbonate (LEDC) or lithium carbonate (Li_2_CO_3_). Both contribute to the passivating effect of the SEI but their stability is still poorly understood. The role of LEDC is unclear, as discussed in the introduction.[^21^, ^22^] We also measured CO at potentials below 0.9 V_Li_, at a rate of 25 pmol s^−1^ mm^−2^ in the first cycle. CO can evolve from the decomposition of either the EC or EMC electrolyte solvents, forming alkoxide species at the SEI.^[49]^ Gasteiger and co‐workers suggested that these alkoxide species trigger continual electrolyte decomposition, further diminishing the cell's lithium ion inventory.^[50]^ In our data, most CO and C_2_H_4_ are evolved in the first cycle. However, a non‐negligible amount is still evolved in the second cycle, indicating that the graphite surface is not entirely passivated after one cycle.

There is some debate in the literature about whether the CO evolution occurs mostly from the decomposition of EC or EMC.[^21^, ^49^, ^51^] C_2_H_4_ is evolved at a rate 2.2 times larger than CO. If it were the case that all CO is evolved from EMC decomposition and all C_2_H_4_ from EC decomposition, then C_2_H_4_ should be evolved at a rate of 0.4 times that of CO (due to the 3 : 7 ratio of EC to EMC in LP57). Therefore, either CO is also evolved from the decomposition of EC or this ratio analysis is not suitable to compare the two solvents. We take the view that it is inappropriate to compare the gas evolution in this way as it does not consider how EC and EMC may interact differently with the graphite electrode—for example EC likely interacts more strongly with graphite due to its greater polarity. Therefore, in order to truly compare the decomposition products of the two solvents, a cell containing an EMC only electrolyte was assembled and measured.

Figure 4b) shows the measured H_2_, C_2_H_4_ and CO signals from a graphite *vs*. Li cell containing 1 M LiPF_6_ in EMC, cycled between 0.01 and 1 V_Li_ for 10 cycles at 1 mV s^−1^. Notably much less H_2_ is evolved in the first cycle, with only one peak observed to begin at around 1.4 V_Li_, coinciding with a peak in the current. Similarly to the LP57 electrolyte, this peak is assigned to trace moisture reduction. Interestingly, no SEI formation resulting in H_2_ evolution is observed from the EMC only electrolyte, consolidating the concept that EMC interacts less strongly with or is more stable to graphite than EC.

CO is measured at a maximum rate of 20 pmol s^−1^ mm^−2^ at potentials below 0.7 V_Li_. This CO is evolved from the reductive decomposition of EMC. Only slightly less CO is evolved in the EMC only cell than the LP57 containing cell, clarifying that most CO is evolved as a degradation product from EMC decomposition. This finding is of importance as it enables the development of better capacity fading models which can now consider the correct number of electrons, solvent molecules and lithium ions lost to each degradation pathway and trapped in the SEI. No ethylene is observed at all, which agrees with the proposed reductive decomposition mechanisms of EMC, which result only in the formation of CO and alkoxide species at the SEI. CO is also measured for the first four cycles, indicating that the SEI formed from EMC is less passivating than the SEI in an EC‐containing electrolyte and a thicker phase and longer formation is required to prevent ongoing electrolyte decomposition (and lithium consumption). This further confirms that the LEDC SEI component performs better than alkoxides.

We also note that the cell performs well for 10 cycles here. This is not possible in most other DEMS and OEMS systems as they require either a flow of a carrier gas through or controlled leak of gas out of the electrochemical cell, resulting in electrolyte drying and limiting measurement lengths.

CO_2_ is observed in significant quantities at the cathode. However the cross‐talk effect of this evolved CO_2_ is not well understood. In order to investigate how CO_2_ influences SEI formation and electrolyte decomposition at the anode, a graphite *vs*. Li cell in 1 M LiPF_6_ in EMC was dosed with CO_2_ via the precise gas handling system in the on‐chip EC‐MS setup. Figure 4c) shows the quantified H_2_, CO_2_ and C_2_H_4_ signals during this measurement. CO_2_ is dosed into the cell for 30 minutes to saturate the electrolyte, after which the backing gas is changed to He. During the CO_2_ dosing, the measured signal is dominated by CO_2_ (which has large fragments at m/z=44 and m/z=28). In order to measure changes in the measured CO_2_ (due to any consumption or evolution) the backing gas was switched back to He once the cell was saturated with CO_2_ and before the electrochemical cycling begun. CO is measured by monitoring m/z=28, however since the cell is saturated with CO_2_, any m/z=28 is attributed to the presence of CO_2_. The CO_2_ signal begins to decay as it is replaced by He. If the CO_2_ were inert, the decay would follow a smooth exponential decay curve: however at about 1 V_Li_, a sharp decrease in the CO_2_ signal is observed, indicating a consumption. This consumption aligns with an increase in the C_2_H_4_ signal, which should not be observed at all in the EMC only electrolyte system (as seen in Figure 4b). This observation provides the first direct evidence to our knowledge of CO_2_ reduction to C_2_H_4_ in a LIB, made possible by the time resolution, sensitivity and dosing capabilities of the novel EC‐MS technique—other studies have investigated the effect of CO_2_ on the graphitic anode but have not been able to observe this reaction.[^50^, ^52^] In aqueous electrolytes, Cu is uniquely active for CO_2_ reduction to the useful chemical C_2_H_4_.^[53]^ It is therefore reasonable to conjecture whether the copper current collector contributes to the unexpected evolution of C_2_H_4_ in a LIB as well. It is also plausible that the reaction is mediated by the presence of plated lithium metal, as observed in nitrogen reduction to ammonia in non‐aqueous systems.^[54]^ The C_2_H_4_ is only observed in the first cycle: we propose that CO_2_ is reduced to solid species in addition to C_2_H_4_, which could explain the stabilising effect of CO_2_ proposed by others in the literature.

Figure 4 c) shows the hydrogen signals evolved and significantly more H_2_ peaks are observed than in the LP57 or EMC‐only systems (although less overall H_2_ is evolved), further highlighting how the presence of CO_2_ increases the graphite anode's reactivity to SEI formation, with multiple processes contributing to H_2_ evolution, rather than just H_2_O reduction.

## Conclusion

To summarise, we have developed a novel operando technique for probing gas evolution in LIBs. On‐chip EC‐MS was adapted for non‐aqueous systems by designing a new electrochemical cell, which enabled us to take advantage of on‐chip EC‐MS's time resolution and sensitivity to probe degradation in LIBs. Finite element method models highlighted the need to design a new electrochemical cell, specific to non‐aqueous electrochemical systems, and demonstrated the electrochemical improvement with the newly designed cell presented in this work.

The value of the technique development was demonstrated by gaining new insight into various LIB degradation mechanisms. The improved time resolution of the technique enabled observation of differences in formation mechanisms of O_2_, CO and CO_2_, evolving from NMC811 cathodes. Importantly, O_2_ evolution from NMC was readily observable in quantities far above the detection limit, unlike other gas detecting techniques, highlighting its suitability for studying more stable, next generation cathode materials. We also note that the precise quantifiability of this work provides critical degradation rates that can validate lifetime prediction models.

We also probed the gas evolving reactions accompanying SEI formation on graphite in a variety of electrolyte systems. We provide evidence that EC reduction dominates C_2_H_4_ evolution and EMC reduction dominates CO formation. This finding should in principle enable the development of better lifetime prediction models. Moreover, the insight provided should enable tailored electrolyte compositions that form SEIs able to prevent continual electrolyte reduction. Future studies should further explore the interaction of various electrolyte solvents (and electrolyte additives) on graphite electrodes with on‐chip EC‐MS, by using isotopically labelled electrolytes or electrodes, for example.

Finally, cross‐talk conditions were simulated by dosing the graphite *vs*. Li cell with CO_2_. A previously undetected reaction pathway was identified, where CO_2_ is reduced to form C_2_H_4_. We hypothesise that CO_2_ reduction to C_2_H_4_ is accompanied by reactions forming solid species in the SEI, which provide stabilising effects. This result also demonstrates the potential of on‐chip EC‐MS for probing gas evolution reactions beyond the realm of battery science,, in the burgeoning field of electrosynthesis in non‐aqueous solvents of high value chemicals.[^55^, ^56^] The newly adapted on chip EC‐MS technique has therefore demonstrated its value in detecting novel degradation mechanisms through its excellent sensitivity, time resolution and ability to simulate cross‐talk conditions.

## Conflict of interest

We have filed a patent on the invention

1

## Supporting information

As a service to our authors and readers, this journal provides supporting information supplied by the authors. Such materials are peer reviewed and may be re‐organized for online delivery, but are not copy‐edited or typeset. Technical support issues arising from supporting information (other than missing files) should be addressed to the authors.

Supporting Information

## Data Availability

Data is available here: https://github.com/dt1414/EC‐MS‐for‐LIBs
