# Supplementary material for: Probing Degradation in Lithium Ion Batteries with On‐Chip Electrochemistry Mass Spectrometry
Source: Angew Chem Int Ed Engl. 2023 Dec 29;63(6):e202315357. doi: 10.1002/anie.202315357 (PMC10962541; doi:10.1002/anie.202315357)
Supplement: Supplementary file 1 — Supporting Information [file ANIE-63-0-s001.pdf]

Angewandte Chemie
Eine Zeitschrift der Gesellschaft Deutscher Chemiker

Supporting Information

Probing Degradation in Lithium Ion Batteries with On-Chip Electrochemistry Mass Spectrometry

*D. B. Thornton, B. J. V. Davies, S. B. Scott, A. Aguadero, M. P. Ryan, I. E. L. Stephens**

1 Technique Comparison

The table below compares On-Chip EC-MS, as developed by SpectroInletes to differential electrochemical mass spectrometry (DEMS) and online electrochemical mass spectrometry (OEMS).

Technique	DEMS	OEMS (Gasteiger)	EC-MS (SpectroInlets)
**Full Name**	Differential Electrochemical Mass Spectrometry	Online Electrochemical Mass Spectrometry	Electrochemistry Mass Spectrometry
**Working Principle**	Differential pumping to protect mass spec from electrochemical environment	Continuous gas head-space sampling from sealed volume	Microfabricated membrane chip with microscopic sampling volume
**Development**	Bruckenstein and Gadde[1]	Tsiouvaras, Gasteiger and coworkers[2]	Trimarco, Vesborg and coworkers[3]
**Approximate Sensitivity (dependent on specie)**	nanomole	Sub-nanomole	picomole
**Collection Type**	Carrier gas, not 100% efficient	Head space sampling	All volatile species collected

**Approximate Response Time**	~1 second	>30 seconds	Sub-second

2 Experimental Details

2.1 EC-MS Cell Assembly

A non-aqueous EC-MS membrane chip (SpectroInlets ApS, described in detail elsewhere[3]) was mounted on to the interface block of the EC-MS equipment and pumped down inside an argon filled glovebox (M-Braun, <0.1 ppm H_2O, <0.1 ppm O_2). A He or CO_2 flow of 1 mL min^{-1} was set to pressurise the sampling volume to 1015 mbar. The non-aqueous cell (depicted in Figure 2 a of main text) was assembled by first placing an O-ring (Kalrez, 3.25mm OD, 1 mm CS) in the cell's stainless steel plunger's O-ring groove. The plunger was then placed in the cell body's central hole. A lithium chip (MTI, 5 mm diameter, 1 mm thick) was then placed on the end of the plunger. A glass microfibre separator (Whatmann GF/C, 6mm diameter, 1mm thick) was placed on top of the lithium. An O-ring (Kalrez, 10 mm OD, 1 mm CS) was placed in the cell body's O-ring groove. The working electrode contact was inserted into its port. In the case of the NMC811 studies, an 8 mm diameter aluminium mesh coated in ~50 µm of NMC811 slurry was placed on top of the stack. In the case of graphite studies, an 8 mm diameter copper mesh, followed by a Celgard 2500 separator coated in the graphite slurry is placed on top of the stack, with the coating facing the mesh. 60 µL of electrolyte (either LP57 or 1M $LiPF_6$ in EMC, Solvionic) was then added to the electrode stack. The cell was mounted on the interface block's clamping ring, ensuring that the current collector was in contact with the working electrode contact. The cell is then closed and fully sealed with a plug.

In the case of the coated separator assembly, an 8 mm diameter separator disk (Celgard 2500) was coated with an anode slurry. The slurry was prepared by mixing 90% graphite (artificial graphite, LiFun), 5% PVDF (Sigma Aldrich) and 5% carbon black (C45, PI Kem) in NMP. This slurry was then cast on to the separator with a doctor blade set to 50 µm and dried on a hot plate at ~100 °C. Once dried, the separator was dried further under vacuum, overnight in the glovebox antechamber at 80 °C, then brought into the glovebox for cell assembly. In the case of the coated mesh assembly, a cathode slurry was prepared by mixing 90% NMC811 (Targray), 5% PVDF (Sigma Aldrich) and 5% carbon black (C45, PI Kem) in NMP. This slurry was then cast on to the mesh with a doctor blade and dried on a hot plate at ~120 °C. Once dried, the mesh was dried further under vacuum, overnight in the glovebox antechamber at 80 °C, then brought into the glovebox for cell assembly.

2.2 Coin Cell Assembly

Coin cells were assembled by cutting 18 mm diameter NMC811 cathodes from a printed electrode sheet (LiFun). The cathode disk was then placed into a positive coin cell casing (MTI), followed by a glass microfibre separator (Whatmann GF/C, 20 mm diameter, 1 mm thick). The separator was then wetted by an electrolyte (LP57, Solvionic). A wave spring and stainless-steel spacer (MTI) were placed into a negative coin cell casing with a gasket (MTI). A lithium chip (MTI, 15 mm diameter) was placed on the spacer. The positive casing containing the cathode and wetted separator was then placed over the negative casing and the cell was crimped shut using an electric crimper (MTI).

2.3 Electrochemical Measurements

The electrochemical measurements were carried out by connecting the cell's contacts to a potentiostat (Biologic SP-200). Galvanostatic measurements were carried out by applying a constant current to the working electrode between an upper and lower cut-off voltage (*vs*. Li). At the upper cut-off voltage, the potential was held until the current dissipated to half the applied current. No such voltage hold was applied at the lower cut-off voltage. Cyclic voltammetry measurements were carried out between an upper and lower cut-off voltage (*vs*. Li) at a scan rate of 1 mV s^{-1}.

The plot below shows an example of a capacity plot, measured for an NMC811 vs. Li experiment. The capacity of the cell on first charge is ca. 150 vs. 130 mAh/$g_{active\ material}$ on discharge, giving a coulombic efficiency (CE) of 86%. First cycle includes additional electrochemical reactions occurring, as observed in the gas evolution in figure 3, due to the formation of the SEI, resulting in a low CE.

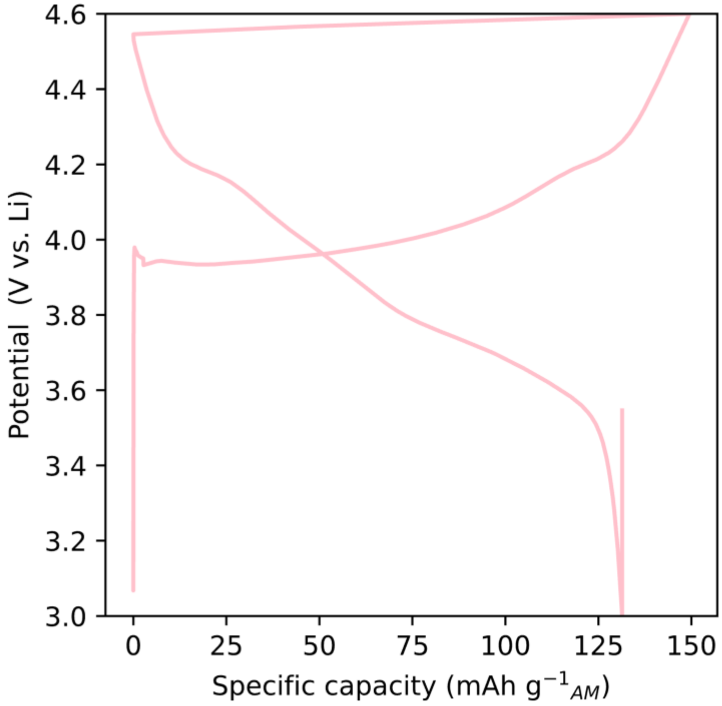

Figure 1: Capacity plot for Figure 3 in the manuscript of the first cycle of NMC811 (WE) vs. Li (CE) in 1M LiPF6 in EC/EMC (3:7), cycled at C/10 as a function of time. The measurements were carried out at 25 +/- 2 degrees Celsius.

2.4 Electrochemistry Mass Spectrometry

Mass to charge ratios of 2, 4, 15, 18, 26, 27, 28, 32, 40 and 44 were monitored for the NMC811 *vs*. Li and graphite *vs*. Li experiments. The respective electrochemical programs were applied to the cell and the mass spectrometry signals recorded throughout. Mass spectra were taken during initial measurements to explore whether any additional species might be present. By performing mass spectra at points of interest throughout the measurement (for example before cycling and at the potential extremes of the measurement), the presence of other gases forming (or not forming) can be monitored. All peaks could be attributed to solvent or analyte molecules so it is highly unlikely that unaccounted for molecules influence the signals at the tracked masses reported.

The open-source python package ixdat was used to read and align the electrochemistry and mass spectrometry data. Mass spectrometry calibration was carried out according to the gas capillary flux method described by Scott[4]. During calibration, a gas of a known composition including an analyte molecule (i) was flowed through the chip. The molar flux of the gas ($\dot{n}^0$) was calculated using the capillary equation

(Equation 3) and multiplied by the mol fraction of the analyte to determine the molar flux of analyte ($\dot{n}^i$). The change in signal at each relevant m/z value due to the analyte flux (S_M) was measured and used to calculate the sensitivity factor, F_M^i, for analyte molecule i at m/z value M, by the Equation 1.

$$F_M^i = S_M/\dot{n}^i \qquad (1)$$

A calibration gas (BOC) with 1000 ppm H_2, C_2H_4, O_2, CO, CO_2 in Ar was used. Note that several analytes with overlapping mass fragments were used in the same calibration gas, requiring the solution of the matrix equation $\vec{S} = \vec{F}\vec{n}$ to obtain the sensitivity factors. During quantification, signals were divided by the determined sensitivity factors to obtain molar fluxes (Equation 2). Once calibrated, a signal background was subtracted. The raw mass spectrometry signals were smoothed with a moving average filter to remove noise with the following factors: CO = 50, CO_2 = 100, O_2 = 100, H_2 = 20. Due to the length of the experiment and timescale of gas evolution, this averaging does not affect the interpretation of the onset potentials for reactions. For the exact calculations, see the scripts in the accompanying GitHub repository. An example of the pre-background subtracted data may also be seen in Figure 1 of the Supporting Information (SI).

$$\dot{n}^i = S_M/F_M^i \qquad (2)$$

3 EC-MS Data Handling

The EC-MS quantification is carried out as described by Scott[1]. In order to determine the sensitivity factor of F_M^i of an analyte i, we must understand its flux through the EC-MS chips's capillary for a given composition of gas in the chip. This capillary flux can be determined by Equation 3 which describes analyte i's flux through three different regimes experienced as the pressure drops from atmospheric pressure to high vacuum. These three regions are a viscous flow regime near atmospheric pressure, a transition regime and a molecular flow regime governed by Kundsen diffusion near high vacuum.

$$\dot{n}_{cap} = \frac{1}{RT}\frac{1}{l_{cap}}\left(\frac{\pi}{8\eta}a^4\bar{p} + \frac{2\pi}{3}a^3\bar{v}\frac{1 + 2\frac{2\sqrt{2}}{\sqrt{\pi}}\frac{a}{\eta}\frac{\bar{p}}{\bar{v}}}{1 + 2.48\frac{2\sqrt{2}}{\sqrt{\pi}}\frac{a}{\eta}\frac{\bar{p}}{\bar{v}}}\right)(p_1 - p_{tran}) + \frac{2\pi}{3}a^3\bar{v}(p_{tran} - p_2)) \qquad (3)$$

Variable	Definition
p_1	Inlet pressure (usually 1 bar)
p_2	Outlet pressure (~ 0)
$p_{tran} = \frac{k_B T}{2\sqrt{2}\pi s^2 a}$	Pressure at which transition from viscous to molecular flow occurs
$\bar{p} = \frac{p_1 + \bar{p}_{tran}}{2}$	Average pressure in viscous flow regime
ν	Viscosity of the gas
$a = \sqrt{\frac{h_{cap} w_{cap}}{\pi}}$	Molecular diameter of the gas
$\bar{v} = \sqrt{\frac{8 k_B T}{\pi m}}$	Mean thermal velocity of the gas molecules
m	Molecular mass
l_{cap}	Capillary length

The raw data collected for this paper can be found in our GitHub repository as well as the associated scripts used to process the data. Figure 1 shows an example of what the quantified data looks like before background subtraction, highlighting that the peaks can clearly be seen and there is no ambiguity in peak identification.

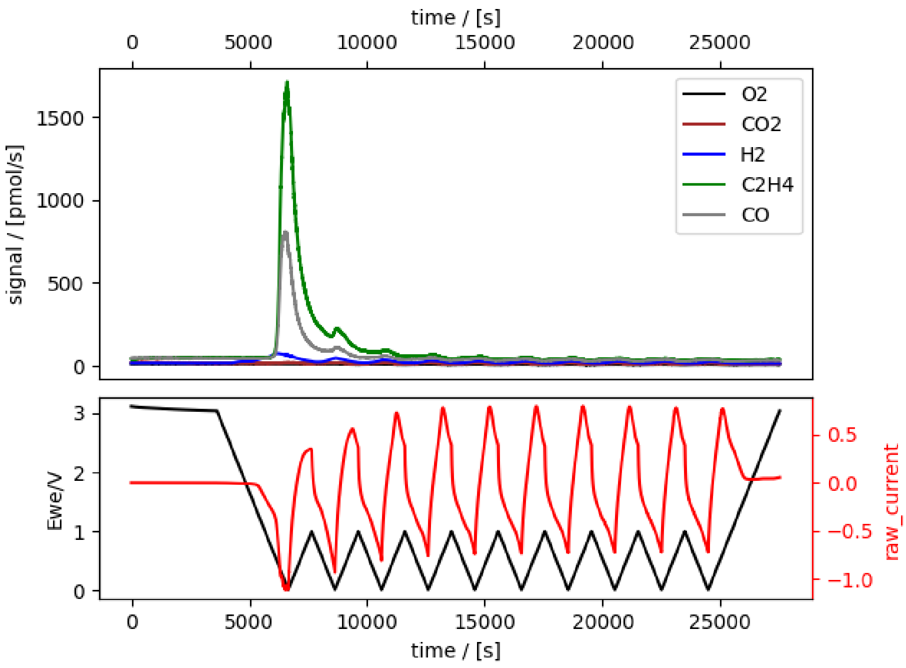

Figure 1: An example plot of quantified data before background subtraction

After smoothing with a moving average filter, the background correction was fitted manually by subtracting a baseline. In most cases this could be done by subtracting a straight line propagated from the flat region of the measured signal before electrochemical cycling takes place, as can be seen in Figure 1. In some cases, where the mass spectrometer's filament had been turned on more recently, a curve had to be fitted to the baseline as the signals stabilised. In this case, again it was clear where the background had to be subtracted, as all signals followed the same decay, and signals that contained peaks were clear. This subtraction was done with a data processing script written in Python which can be found in this work's Github repository.

Our cell was assembled and mounted onto the chip and mass spectrometer inside a glovebox. We began our measurements before mounting the cell. As the chip is exposed to the glovebox environment, the mass spectrometer measures a strong argon signal. Once the cell is mounted, if there is no leakage, the argon signal decreases steadily as the argon saturated electrolyte becomes saturated with the helium from the backing gas instead. Both argon and helium may be used as a backing gas due to their inert nature, however using helium as the backing gas enables the user to confirm a tight cell with no leaks (any increase in argon would signify a leak).

The calibration measurement similarly took place with a tightly sealed environment, where a glass plate was mounted on top of the mass spectrometer inlet rather than a cell and chip. This enabled us to measure the flow of the backing gas (which in the case of the calibration measurement was the calibration gas), and

the backing gas only. Since the quantity desired is a molecular flux in pmol per second, the flux of the calibration gas through the capillary during the calibration experiment is calculated. The ratio of the measured signal during the calibration measurement to this flux is used as the sensitivity factor (see Trimarco *et al.*[3] for further details). Such calibration experiments were performed for each species. However, to account for changes in the overall mass spectrometer sensitivity (attributed to changes in the filament's ionising ability over time), we also measured the mass spectrometer's sensitivity to the glovebox environment's argon at the time of the calibration measurement. During quantification of a measurement, the pre-calculated sensitivity factors are scaled by comparing the glovebox argon signal from the measurement to the glovebox argon signal from the calibration. This removes the requirement to perform a new calibration measurement for each experiment. All of the practical steps are provided in the quantification codes in this work's Github repository.

4 Cell Design

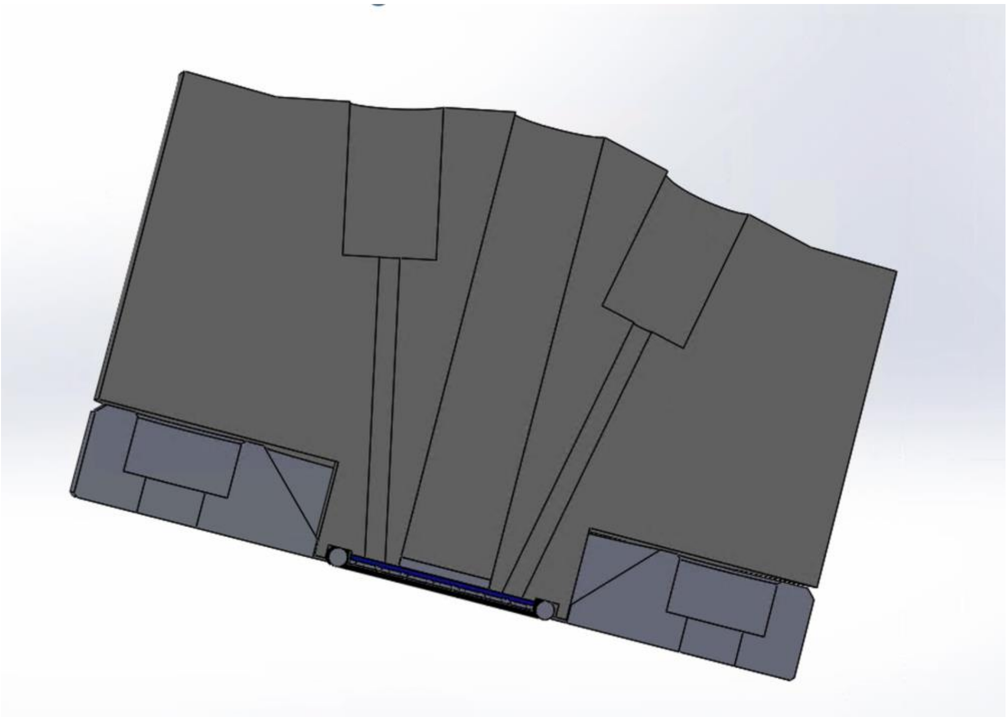

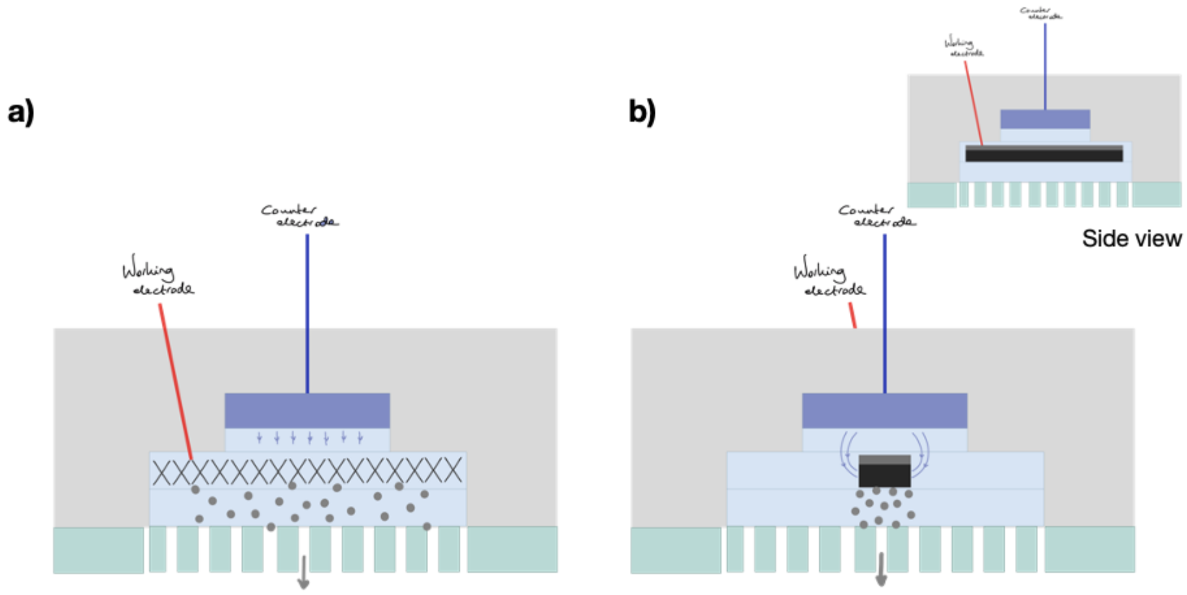

Figure 2: Above: A computer aided design drawing of the newly designed EC-MS cell. Below: The two electrode configurations the cell can be assembled in. a) shows the mesh current collector configuration and b) shows the strip electrode configuration.

There are two possible electrode configurations in the designed cell. One configuration employs a mesh current collector coated in the working electrode slurry (depicted in Figure 2 a). The other configuration employs a strip electrode that must be contacted to the working electrode contact (depicted in Figure 2 b). The strip configuration employs a typical planar working electrode that has been cast on to a foil current collector. In this configuration, the electrode is cut into a rectangular strip and surrounded by separator wetted by an electrolyte. In this case, the electrode coating faces the membrane and the current collector faces the counter electrode. During electrochemical cycling in a LIB, lithium ions must diffuse around the current collector into the active electrode coating and gases evolved will diffuse downwards towards the membrane. Figure 2 describes and compares the two configurations. This strip configuration's lithium ion diffusion pathway is longer and less direct than the mesh configuration which is detrimental to the electrochemical performance of a cell. However, the ability to operate in this configuration means that electrodes from commercial cells can be studied, even from aged cells. We note that this configuration would also facilitate measurements from model, thin-film electrodes.

5 Finite Element Method Modelling

5.1 Method

The Battery Design module of COMSOL Multiphysics (5.6) was used to build a 2-dimensional finite element model of the operation of the aqueous and non-aqueous cell geometries (available in our GitHub repository). The model was based on the pseudo-2D Newman model[2]. The cells implemented 1M $LiPF_6$ in ethylene carbonate/ethyl methyl carbonate (3:7) as the electrolyte. They also both implemented a porous working electrode with a matrix made up of 40% NMC811, 40% electrolyte and 20% carbon/binder. Lithium metal was set as the counter electrode for both cell geometries.

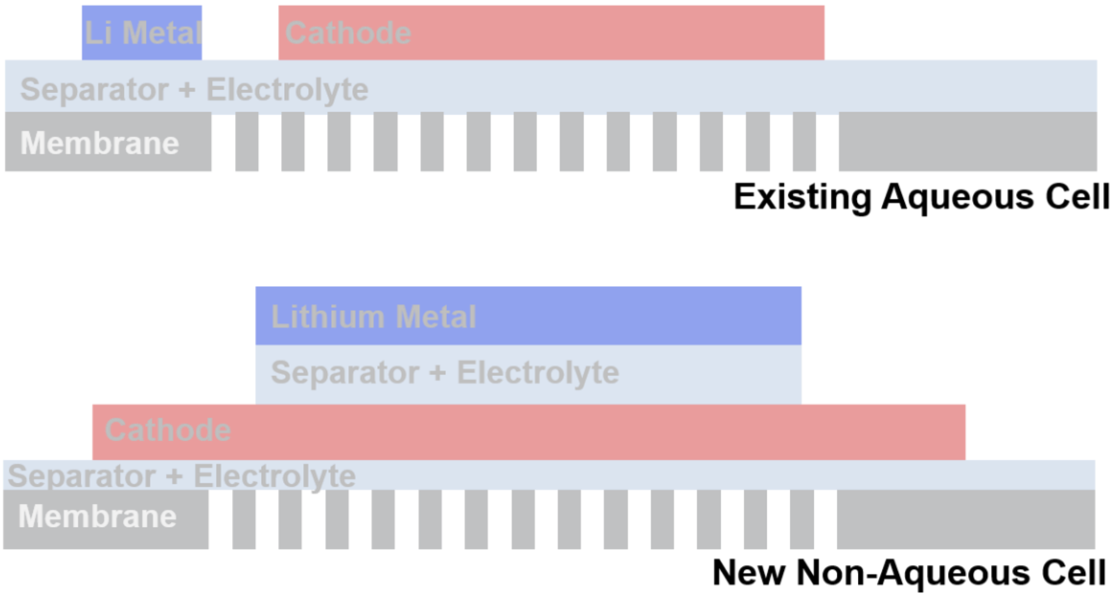

Figure 3: The two cell geometries that the operation of a LIB was explored in. In the existing aqueous cell geometry, the cathode and lithium metal electrodes are arranged side by side and in the new non-aqueous cell geometry the electrodes are parallel.

Operation of a LIB was explored in two cell geometries: the existing aqueous and the new non-aqueous cell. These geometries are depicted in Figure 3. In the aqueous cell geometry, the counter electrode is a 1 mm diameter lithium metal located 1 mm away from the 5 mm diameter NMC811 cathode. A 100 µm thick layer of wetted separator ionically contacts the two electrodes. In the new non-aqueous cell geometry, the counter electrode is a 5 mm diameter lithium metal disc placed parallel and centrally above an 8 mm diameter NMC811 cathode. A separator wetted by the electrolyte is placed above and below the cathode, ensuring ionic contact.

The Transport of Dilute Species module of COMSOL Multiphysics (5.6) was used to build a 2-dimensional finite element model to describe the mass transport of evolved volatile species in the new electrochemical cell (available on our GitHub repository). The model considers the Fickian diffusion of the volatile species through the cell once evolved. Diffusion coefficients were determined via the Stokes-Einstein Relation. The model also considers the solutes' Henry's Law constants to describe their evaporation into the sampling volume. These values were calculated from the electrolyte viscosities and solubilities determined by Dougassa *et al*.[6] The basis for this model is described in more detail elsewhere[4]. Diagrams representing the cell geometries through which the volatile species are transported through can be seen in Figure 4.

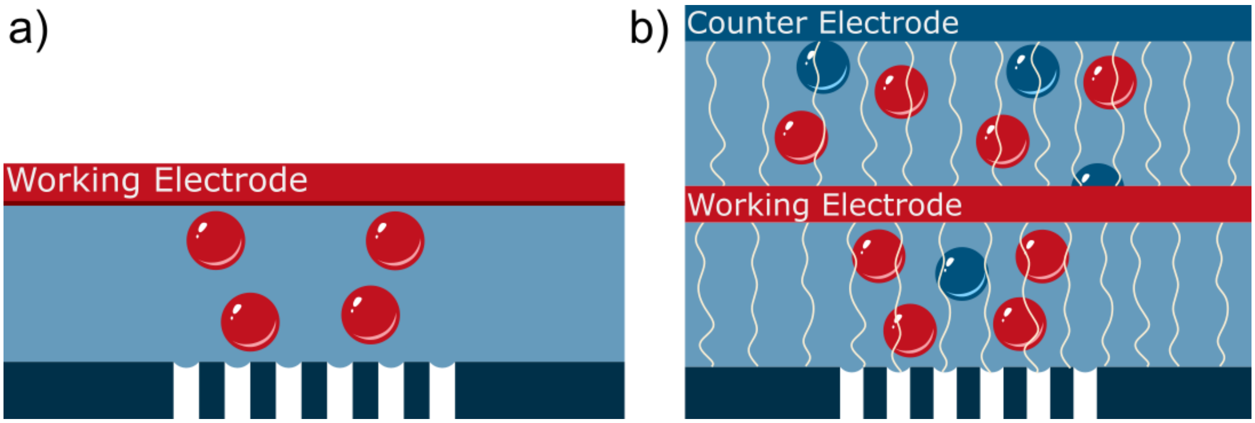

Figure 4: The two configurations explored by the mass transport models. a) depicts the configuration where a constant molar flux is evolved from a planar working electrode, and no separators are located between the working electrode and the membrane. b) depicts the configuration where a constant molar flux is evolved isotropically from a working electrode volume. In this configuration separators may be implemented as well as gas evolution from a counter electrode.

The mass transport models explore a few configurations. The basic configuration to compare differences in time-response between a variety of electrolytes is depicted in Figure 4 a. In this configuration a constant molar flux of analyte evolves from a planar surface on the working electrode. The species diffuses towards the membrane chip, establishing a concentration gradient in the electrolyte. At the membrane, the analyte evaporates into the chip's gas phase sampling volume. Figure 4 b depicts the model configuration that investigates the effect of the porous separators on the mass transport. In this configuration the analyte evolves isotropically from a working electrode volume. Again, the analyte establishes a concentration gradient in the cell space and evaporates at the membrane, into the chip. The properties of the separators may be the same or different and a constant molar flux of analyte may also be set to evolve from the counter electrode. The mass transport COMSOL model files may be found in our GitHub repository.

The model built to describe the mass transport of volatile species in the electrochemical cell takes into consideration three transport processes once a constant flux of analyte is evolved from a surface or volume: the diffusion through the working volume, mass transfer across the liquid-gas interface at the chip surface and the molar flux of gas through the chip capillary towards the mass spectrometer. The diffusion through the working volume (electrolyte or electrolyte and separator) can be described by Fick's second law (Equation 2), where c is the concentration, t is time and D is the diffusion coefficient of the analyte in the solvent. The diffusion coefficients are determined via the Stokes - Einstein Relation (Equation 3), where k_B is the Boltzmann constant, T is the temperature and a is the molecular bond length. In the case of the models that investigated the effect of the separators on the mass transport, the diffusion coefficients were modified *via* the Bruggeman correlation (Equation 4) which defines an effective diffusion coefficient that takes in to consideration the porosity, ϵ, and tortuosity, τ, of the separators.

$$\frac{\partial c}{\partial t} = D\frac{\partial^2 c}{\partial x^2} \qquad (2)$$

$$D = \frac{k_B T}{6\pi \nu a} \qquad (3)$$

$$D_{eff} = D\frac{\epsilon}{\tau} \qquad (4)$$

The analyte species' mass transfer from the liquid environment of the cell into the gas environment of the chip is dependent on the species' volatility and can be defined by a mass transfer coefficient h (Equation 5). The coefficient h is defined by the molar flux density of the analyte across the interface (j) divided by the concentration (c) of the analyte at that point on the interface. This coefficient may be defined as a constant that takes in to account the species' Henry's Law Constant (K_H), the gas constant (R), the temperature (T), the flux to the mass spectrometer (Q_0), the pressure behind the membrane (p_m) and the membrane area (A_m). For further details on this definition, refer to Scott et al.[4]

$$h = \frac{j(x,y)}{c(x,y,t)} = K_H\frac{RTQ_0}{p_m A_m} \qquad (5)$$

The molar flux through the capillary happens at timescales orders of magnitudes faster than the diffusion and evaporation processes and so its effects are assumed to be negligible and it is not considered here. The model solves Fick's first law (Equation 6) at the membrane interface and calculates the flux into the chip.

$$D\frac{\partial c}{\partial x} = hc \qquad (6)$$

5.2 Electrochemical Improvements with the New Cell Design

A 2-dimensional finite element model was built with COMSOL Multiphysics (5.6) to compare the operation of the existing aqueous electrochemical cell and new non-aqueous electrochemical cell geometries in LIB conditions.

The lower panel in Figure 5 shows the constant current of 0.01 A that was applied at the cathode to an upper cut-off voltage of 4.4 V_{Li}. -0.01 A was then applied at the cathode to discharge the cell to 3.0 V_{Li}. The average electrode potential was calculated and plotted in the upper panel. This panel in Figure 5 shows that the existing aqueous cell has a significantly higher over potential than the new non-aqueous cell. Additionally, the very large drop in potential (~0.7 V_{Li}) at the beginning of the second discharge indicates a very high internal resistance that is not present in the non-aqueous cell. These effects are so detrimental in the aqueous cell, that the full state of charge (SOC) range (24%-100%, defined by the model) was not accessible (as can be seen in the middle panel of Figure 5). The new non-aqueous cell geometry could however achieve the full range. Upon increasing the applied current, the effect became worse, to the point where the model was unable to converge as the rate of lithium mass transport couldn't satisfy the applied current density. To further investigate the source of the overpotential and internal resistance, the local state of charge and potential distributions were calculated.

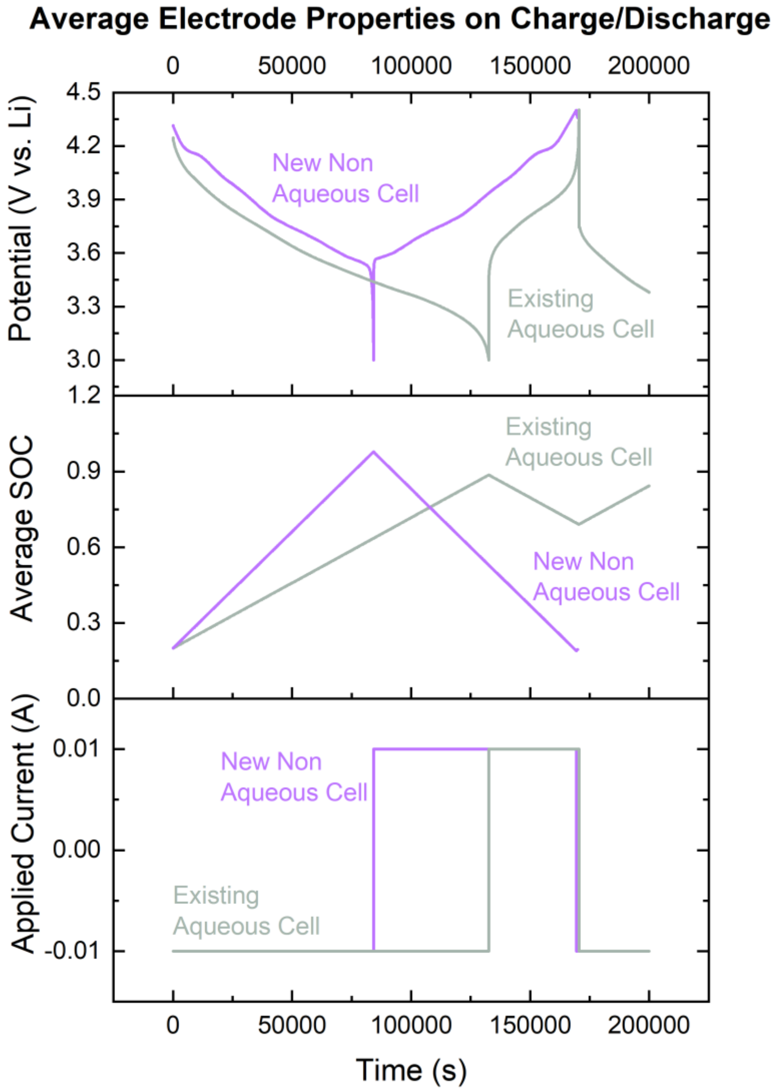

Figure 5: The applied current (lower panel), calculated average electrode state of charge (middle panel) and calculated potential (upper panel) in the existing aqueous EC-MS cell and the new non-aqueous EC-MS cell geometries (both NMC811 *vs*. Li). The new non-aqueous cell performs far better than the aqueous cell, with no internal resistance contributions and avoiding the over potential measured in the aqueous cell.

Figure 6 a) and b) show the state of charge distribution across the aqueous and non-aqueous working electrodes respectively as a function of the galvanostatic cycling described above. The blue lines indicate the local state of charge of the left side of the working electrodes in the aqueous (Figure 6 e) and non-aqueous (Figure 6 f) cells, as highlighted by the blue stars. The red lines indicate the local state of charge on the right hand side of the working electrodes in the aqueous (Figure 6 e) and non-aqueous (Figure 6 f) cells, as highlighted by the red stars. There is a large discrepancy between the state of charge experienced

on the left and right sides of the electrode in the existing aqueous cell - up to 50% difference at times. The cell geometry kinetically limits the electrode's uptake of lithium ions, resulting in this severe state of charge inhomogeneity. This inhomogeneity will induce different gas evolution behaviour throughout the electrode, rendering any gas evolution measurements as a function of electrochemical cycling from the aqueous cell, completely inaccurate. The new non-aqueous cell does not experience any difference between the left and right sides of the electrode above the membrane. The state of charge homogeneity of the new non-aqueous cell therefore corroborates the EC-MS measurement results.

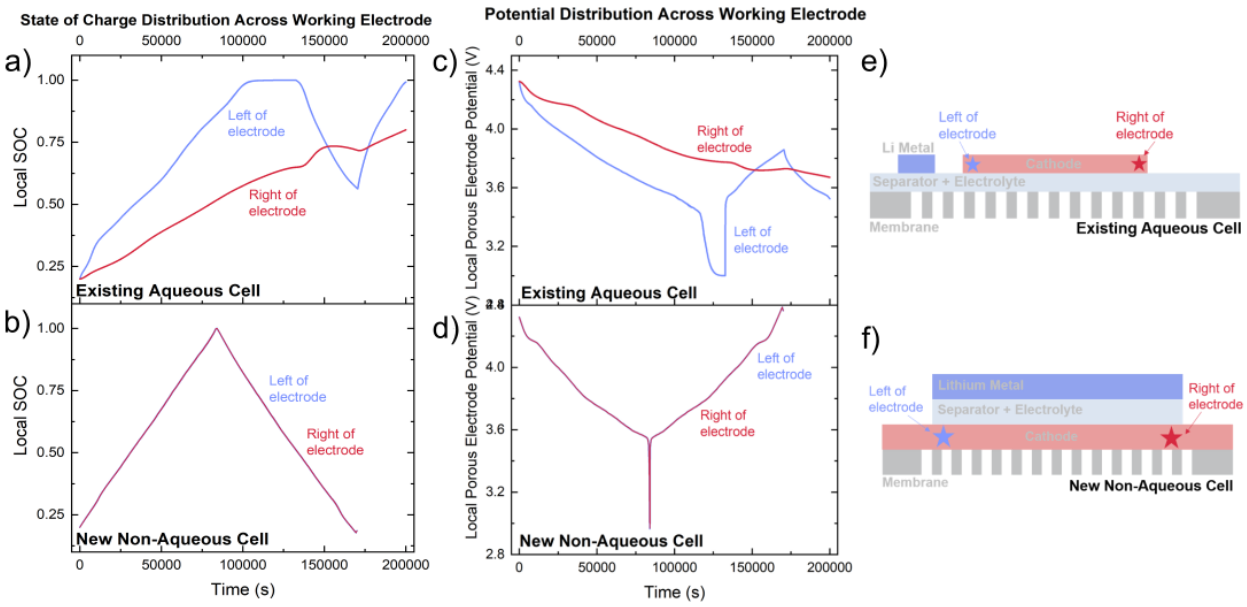

Figure 6: The calculated a) local state of charge in the aqueous cell, b) local state of charge in the non-aqueous cell, c) local electrode potential in the aqueous cell and d) local electrode potential in the non-aqueous cell during the constant current charge and discharge. e) indicates the locations in the aqueous cell geometry where the local electrode properties were calculated at and f) indicates the locations in the aqueous cell geometry where the local electrode properties were calculated at - the blue indicates the left side of the NMC811 porous working electrode and the red indicates the right side. The new non-aqueous cell exhibits homogeneous potential and state of charge distribution whereas the aqueous cell exhibits severe inhomogeneity.

Figure 6 c) and d) show the calculated potential distribution across the aqueous and non-aqueous working electrodes as a function of the galvanostatic cycling described above. The blue lines indicate the local electrode potential measured on the left side of the working electrodes in the aqueous (Figure 6 e) and non-aqueous (Figure 6 f) cells, as highlighted by the blue stars in the diagrams below. The red lines indicate the local electrode potential measured from the right side of the working electrodes in the aqueous (Figure 6 e)

and non-aqueous (Figure 6 f) cells, as highlighted by the red stars in the diagrams. Again, we observe a significant inhomogeneity in the electrode potential distribution. Similarly to the state of charge, an electrochemical system's gas evolution behaviour is heavily dependent on potential. The variation in potential throughout the aqueous cell's electrode leads to enormous uncertainty, making the cell a poor approximation of a real battery. The new non-aqueous cell exhibits a homogenous potential distribution throughout its working electrode, representative of a real battery, where the electrodes are parallel to each other. By implementing the new cell, the time sensitivity of the membrane can be exploited to extract accurate and reliable data about potential-dependent gas evolution.

Gif animations showing the local state of charge in the aqueous and new non-aqueous cells can be found in the GitHub repository. The COMSOL files containing the models and parameters used can also be found in the repository.

5.3 Mass Transport of Volatile Species

We investigated the mass transport of various volatile species in electrolyte systems in the new non-aqueous cell. Figure 7 a) shows the predicted quantified mass spectrometer signal for a 100 second pulse of CO_2 evolved from the working electrode at an arbitrary but realistic rate of 0.00013 mol m^{-2} s^{-1} in various electrolyte systems. These calculations consider the gas evolution from the working electrode surface and their transport through an electrolyte directly towards the chip, without any separators (as depicted in Figure 4 a). The time response to observe any signal once the electrode evolves CO_2 is almost instantaneous (sub second). This is a significant improvement over most DEMS and OEMS systems which often have time responses on the order of minutes to detect a gas once it is produced in the cell. This improvement is crucial for determining accurate onset potentials, as well as improving the detectability of species before they react with other components in the cell. We note here that this excellent time response is even observed with CO_2, a relatively slow molecule, when compared to H_2 for example. For all electrolyte systems, the time response to reach equilibrium is on the order of tens of seconds, with some variability dependent on the salt concentration and solvent system. This time resolution is dependent on the electrolyte viscosity and Henry's Law constants of the analytes in the electrolyte. The model's results allow us to tailor the electrolyte composition to suit the aim of the experiment: for example if we wanted to observe especially short lived species, we might opt for a very low concentration $LiPF_6$ based electrolyte in EC/DMC. Similarly, if the goal was to minimise the effects of cross-talk, we might choose a high concentration LiFAP in EC/DMC solution. This analysis was also carried out for O_2, CO and C_2H_4 (Figure 8 and Figure 9).

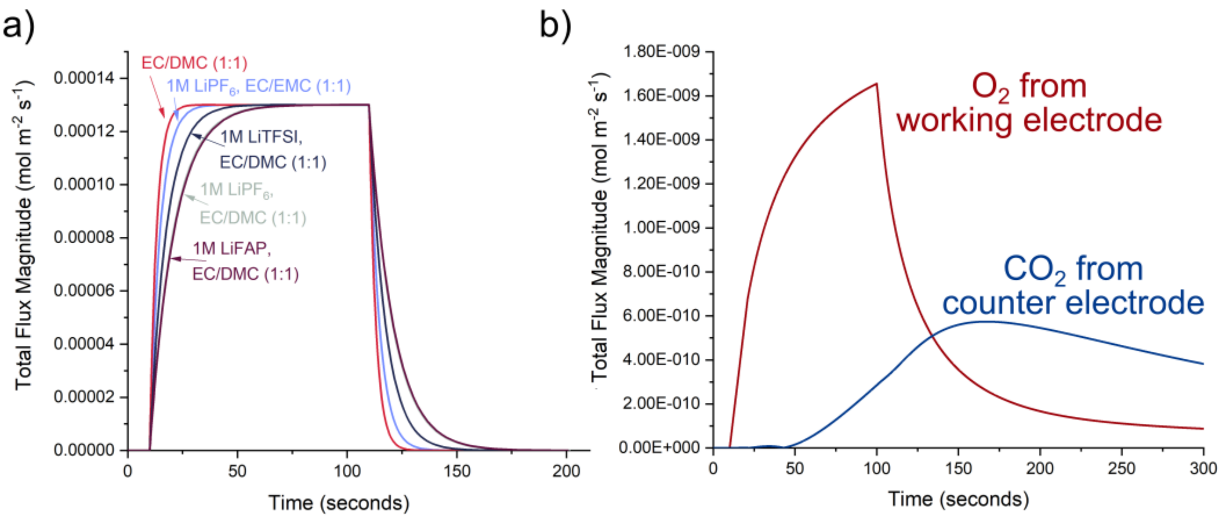

Figure 7: a) The predicted mass spectrometer signals for a 100 second pulse of CO_2 evolving in the non-aqueous electrochemical cell in a variety of electrolyte systems. Although all systems enable instant detection of the CO_2, some electrolyte formulations display different time responses. b) The predicted mass spectrometer signals for a 100 second pulse of CO_2 evolving from the counter electrode and O_2 evolving from the working electrode in the non-aqueous electrochemical cell. Although both species are detectable, only the working electrode signal is sharp, facilitating the deconvolution of peaks.

To consider the effect of using different separators in the electrochemical cell we altered the diffusion coefficients of the species via the Bruggeman correlation. The Bruggeman correlation considers the porosity and tortuosity[7] of the material being wetted by the electrolyte, calculating an effective diffusion coefficient. The model was also developed to include the two separators above and below the working electrode: one in between the working and counter electrodes and one in between the working electrode and the chip (as depicted in Figure 4 b). In this model, the volatile species are set to diffuse isotropically from an electrode volume, rather than from one electrode surface as in the previous mass transport model. This modification confirmed that using a less porous separator above the working electrode and a more porous electrode between the working electrode and the chip improves the time resolution when compared to a cell that only uses the less porous separator in both positions (Figure 10). We also predicted the quantified mass spectrometer signals from two different species being evolved simultaneously in the same quantity from the working and counter electrodes. Figure 7 b shows that the O_2 evolved from the working electrode increases sharply as expected. Figure 7 b also shows that the presence of the separator does not have significant implications on the time resolution since the porous separator (Celgard 2500) is used between the membrane chip and working electrode. The CO_2 signal evolved from the counter electrode is however severely slowed as it must diffuse through the less porous separator (Celgard 2325) as well as the working electrode and more porous electrode before it reaches the mass spectrometer. This is a valuable

finding as it allows us to rule out any sharp peaks as being evolved from the counter electrode, enabling deconvolution of signals and allowing us to assign which electrode the measured species originates from.

The mass transport properties were explored for O_2, C_2H_4 and CO as well as the calculated properties for CO_2 (presented above). Figure 8 shows the calculated mass spectrometer signals for O_2, C_2H_4, CO_2 and CO in a variety of electrolyte species. We note the significant variability in the time responses in the various electrolyte systems. This data is valuable for design of experiments as well as aiding interperability of experimental data.

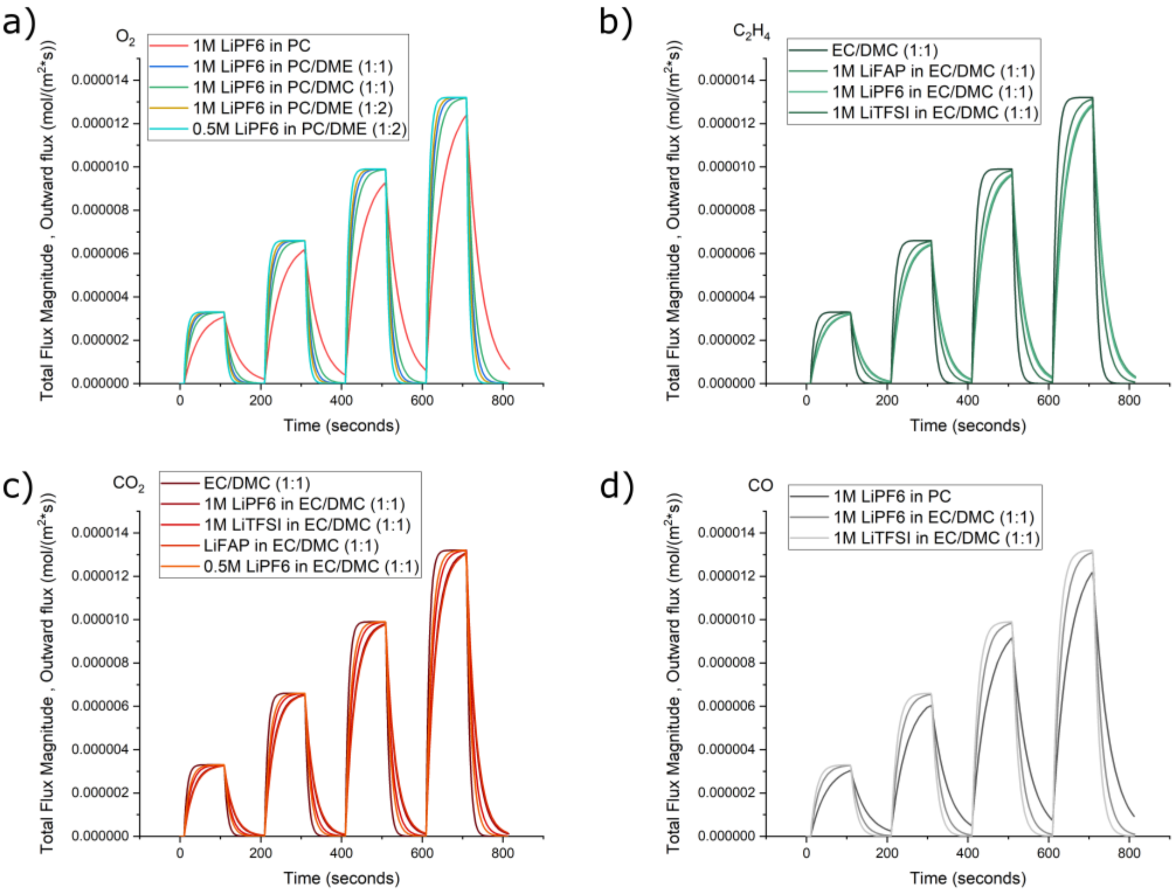

Figure 8: The predicted mass spectrometry signals for four constant pulses of evolved analyte from a planar working electrode surface. The pulse magnitudes are set to increase with each pulse. Each panel represents the predicted signals for different analytes in a variety of electrolyte systems where panel a) shows the O_2 flux, b) shows the C_2H_4 flux, c) shows the CO_2 flux and d) shows the CO flux.

Figure 9 compares the calculated mass spectrometer signals for C_2H_4, CO, CO_2 and O_2 in 1M $LiPF_6$ in EC/DMC (except in the case of O_2 where the system is 1M $LiPF_6$ in PC/DMC due to data availability). We

can see that C_2H_4 is the slowest of these species, followed by CO, CO_2 and then O_2. Again, this understanding is of great value when interpreting experimental data.

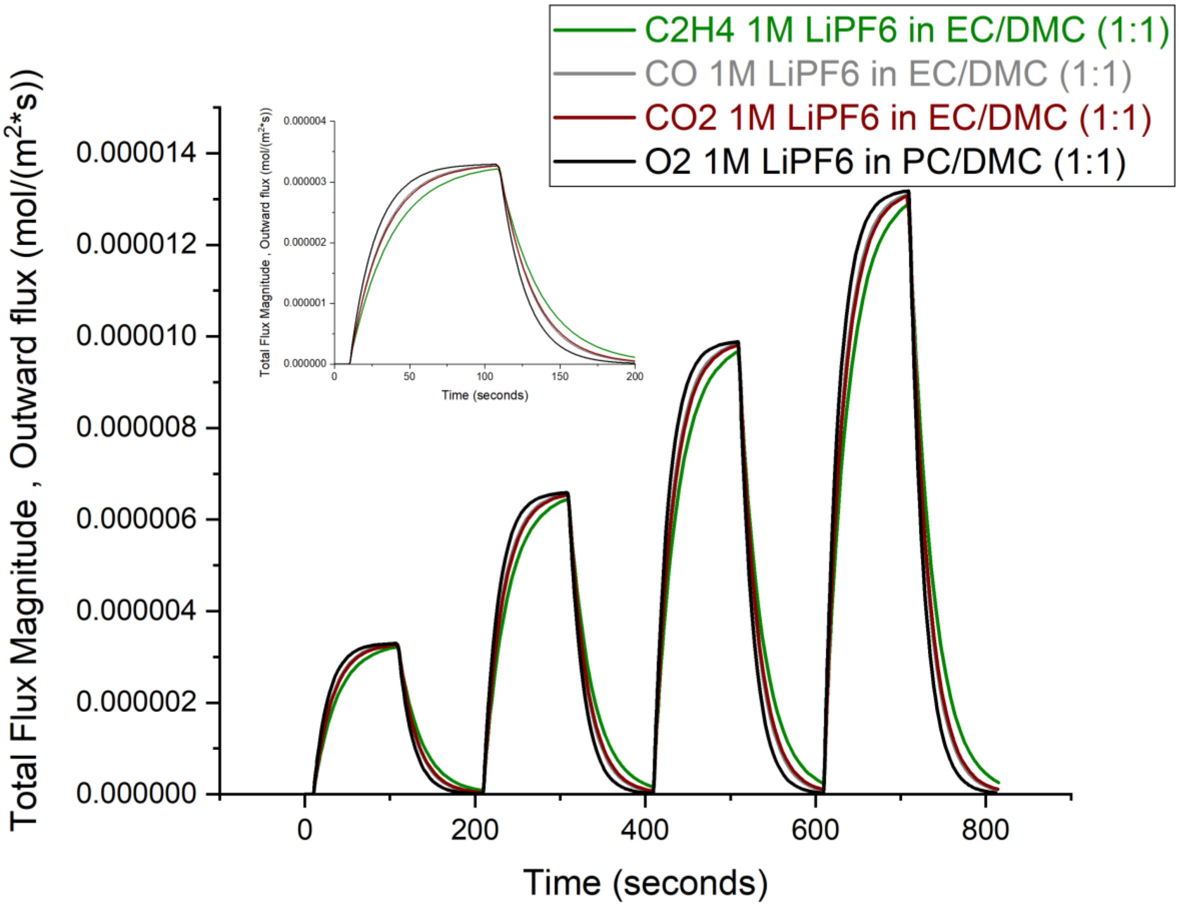

Figure 9: The calculated mass spectrometer signals for four pulses of evolved C_2H_4, CO, CO_2 and O_2 in a 1M $LiPF_6$ electrolyte system from a planar working electrode surface. The inlay shows the first pulse only.

How the mass transport properties are influenced by the separators was also investigated. Figure 10 shows the calculated mass spectrometer signals for a constant molar flux of O_2 being evolved from a working electrode volume in 1M $LiPF_6$ in EC/PC (1:1) for two different separator systems. In one system, Celgard 2500 (the more porous separator) is used both above and below the working electrode, labelled same porosity. In the other system, the more porous Celgard 2325 is used below the working electrode. The calculation shows that using this more porous electrode below the working electrode does indeed improve the time resolution of the system as the volatile species are more likely to diffuse towards the membrane through the less tortuous and more porous path.

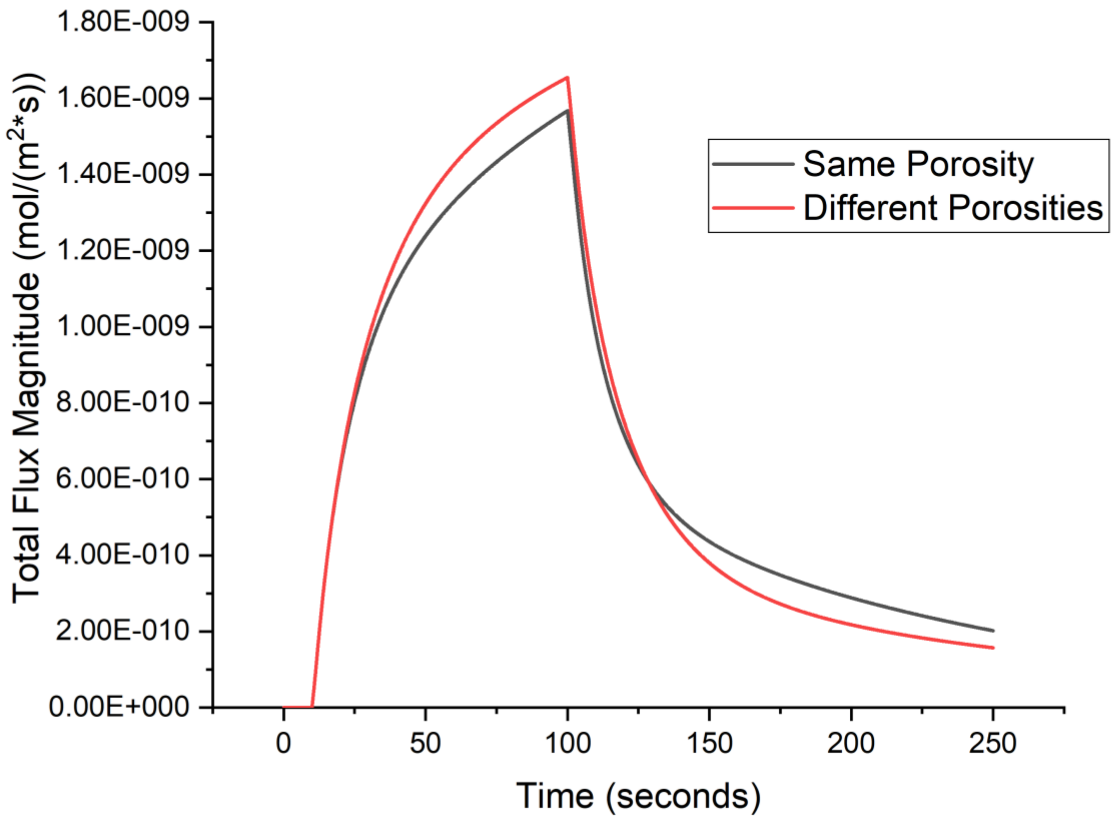

Figure 10: Comparison of a constant molar flux of O_2 being evolved from a working electrode volume where Celgard 2500 is used above and below the working electrode (same porosity) and where Celgard 2325 is used above and Celgard 2500 is used below (different porosities). The time resolution of the system is improved by employing a less porous separator above the working electrode.

All Henry's Law constants, viscosities, calculated diffusion coefficients and calculated mass transfer coefficients are presented in Table 1. The viscosities and Henry's Law constants were collected from Dougassa *et al.*[8,9]

Table 1: Constants used for mass transport model for analytes in various electrolytes

**Solution**	**K_H (Mpa)**	**Viscosity (mPa s)**	**D**	**h**
C2H4				
EC:DMC (1:1)	12.53	1.192	4.403999E-10	7.03E-05

Solution	K_H (Mpa)	Viscosity (mPa s)	D	h
1M LiFAP in EC:DMC (1:1)	12.62	4.25	1.235192E-10	7.08E-05
1M LiPF6 in EC:DMC (1:1)	9.48	4.152	1.264347E-10	5.32E-05
1M LiTFSI in EC:DMC (1:1)	9.75	2.888	1.817717E-10	5.47E-05
1M LiTFSI in EC:PC:DMC (1:1:3)				
1M LiPF6 in EC:PC:DMC (1:1:3)				
CO				
1M LiPF6 in EC	274			
1M LiPF6 in PC	146.7	8.06	8.493586E-11	8.23E-04
1M LiPF6 in DMC	99.7			
1M LiPF6 in EMC	82.1			
1M LiPF6 in DEC	71.9			
1M LiPF6 in EC:DMC (1:1)	151.7	4.152	1.648803E-10	8.51E-04
1M LiTFSI in EC:DMC (1:1)	138.4	2.888	2.370440E-10	7.76E-04
1 M LiPF6 in EC:PC:DMC (1:1:3)	125.1			
1M LiTFSI in EC:PC:DMC (1:1:3)	116.2			
CO2				
EC:DMC (1:1)	10.2	1.192	5.485221E-10	5.72E-05
1M LiPF6 in EC:DMC (1:1)	11.76	4.152	1.574755E-10	6.60E-05
1M LiTFSI in EC:DMC	10.2	2.888	2.263983E-10	5.72E-05
1M LiFAP in EC:DMC (1:1)	10.42	4.25	1.538443E-10	5.85E-05
0.5M LiPF6 in EC:DMC (1:1)	11.24	2.243	2.915017E-10	6.31E-05
O2				
1M LiPF6 PC:EC (1:1)	20.74689	7.73	9.675078E-11	1.16E-04
1M LiPF6 PC	19.37984	8.06	9.278952E-11	1.09E-04

Solution	K_H (Mpa)	Viscosity (mPa s)	D	h
1M LiPF6 PC:DME (1:1)	13.85042	2.59	2.887581E-10	7.77E-05
1M LiPF6 PC:DMC (1:1)	13.71742	3.50	2.136810E-10	7.70E-05
1M LiPF6 PC:DEC (1:1)	12.70648	4.78	1.564610E-10	7.13E-05
1M LiPF6 PC:DME (1:2)	10.02004	1.98	3.777190E-10	5.62E-05
0.5M LiPF6 PC:DME (1:2)	8.210181	1.19	6.284736E-10	4.61E-05

The other constants required to calculate the diffusion coefficients and mass transfer coefficients are presented in Table 2. The separator constants were collated from Finegan *et al.*[4]

Table 2: Constants required for mass transport model calculations

Constant	Value	Unit
K_b	1.380649E-23	$m^2\ kg\ s^{-2}\ K^{-1}$
T	298.15	K
R	8.314463	$J\ K^{-1}\ mol^{-1}$
Q_0	8.60E-09	$mol\ s^{-1}$
A_m	3.80E-05	m^2
P_m	1.00E+00	bar
a (O_2)	2.92E-10	m
a (CO_2)	3.34E-10	m
a (CO)	3.19E-10	m
a (C_2H_4)	4.16E-10	m
ϵ (Celgard 2325)	0.41	
ϵ (Celgard 2500)	0.53	
τ (Celgard 2325)	2.23	
τ (Celgard 2500)	1.43	

6 Additional Analysis of NMC811 vs. Li EC-MS Data

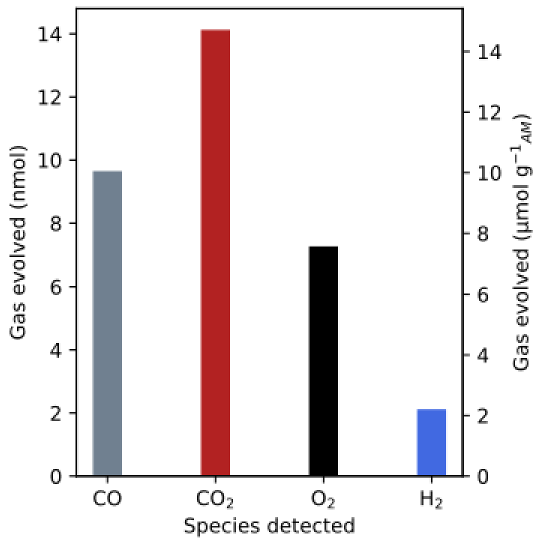

Figure 11: The integrated total amounts of gases evolved in the above EC-MS measurement of a NMC811 *vs.* Li cell, for one cycle carried out at C/10 in LP57 at room temperature.

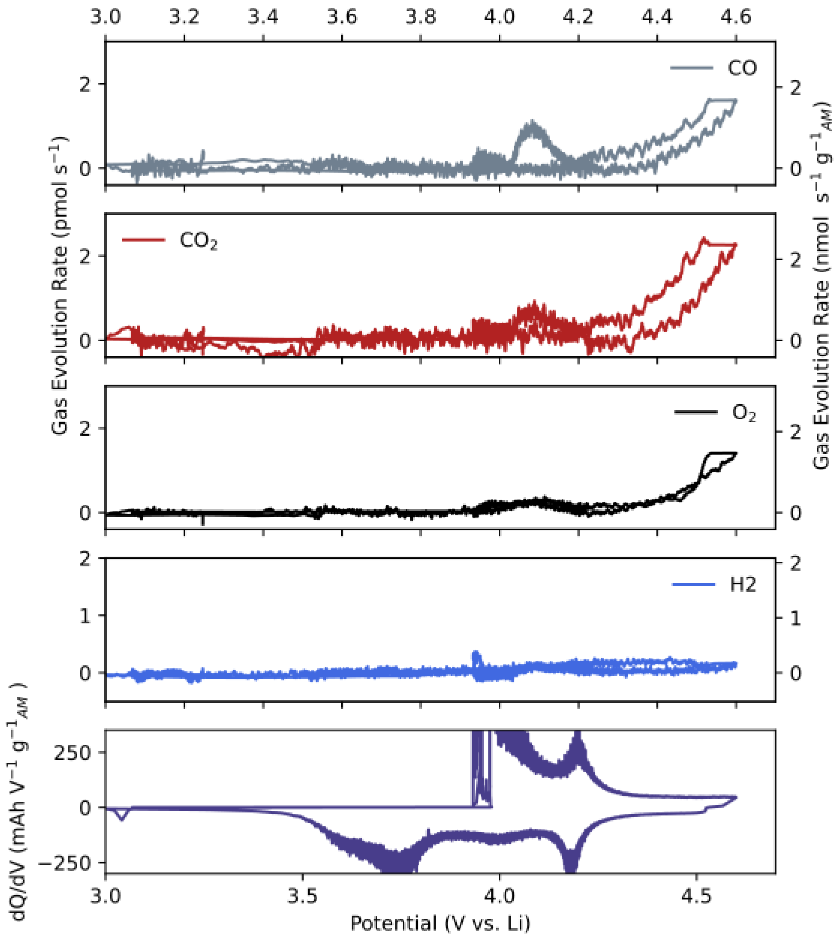

Figure 12: The measured H_2, O_2, CO_2 and CO signals from a NMC811 *vs*. Li cell, cycled at C/10, in LP57 at room temperature, plotted as a function of potential, highlighting the relationship between phase changes and gas evolution.

7 Summary of Contents in GitHub Repository

A Github repository containing useful code and model files may be found here: https://github.com/dt1414/EC-MS-for-LIBs

- COMSOL Electrochemistry Models
  - COMSOL model files

    - Gifs showing local state of charge of the working electrode in the aqueous and non-aqueous cell geometries
- COMSOL Mass Transport Models
    - COMSOL model files
- EC-MS Data
    - Raw calibration data
    - Graphite vs Li in LP57 (raw data files and processing scripts)
    - Graphite vs Li in EC-Free (raw data files and processing scripts)
    - Graphite vs Li in EC-Free + CO2 (raw data files and processing scripts)

References

References in table:

1. S. Bruckenstein, R. Gadde, *J. Am. Chem. Soc.*, 93, 3, 793–794 (1971)
2. N. Tsiouvaras, S. Meini, I. Buchberger, H. A. Gasteiger, *J. Electrochem. Soc.* **160** A471 (2013)
3. D. B. Trimarco, S. B. Scott, A. H. Thilsted, J. Y. Pan, T. Pedersen, O. Hansen, I. Chorkendorff, and P. C. K. Vesborg, *Electrochimica Acta*, 268, 520–530 (2018)
4. S. B. Scott, thesis, (2019)
5. M. Doyle, T. F. Fuller, and J. Newman, *Journal of The Electrochemical Society*, **140**, 1526–1533 (1993)
6. Y. R. Dougassa, J. Jacquemin, L. E. Ouatani, C. Tessier, and M. Anouti, *Journal of Physical Chemistry B*, **118**, 3973–3980 (2014)
7. D. P. Finegan, S. J. Cooper, B. Tjaden, O. O. Taiwo, J. Gelb, G. Hinds, D. J. L. L. Brett, and P. R. Shearing, *Journal of Power Sources*, **333**, 184–192 (2016)
8. Y. R. Dougassa, C. Tessier, L. E. Ouatani, M. Anouti, and J. Jacquemin, *Journal of Chemical Thermodynamics*, **61**, 32–44 (2013)
9. Y. R. Dougassa, J. Jacquemin, L. E. Ouatani, C. Tessier, and M. Anouti, *The Journal of Chemical Thermodynamics*, **79**, 49–60 (2014)
